# Bridging hospital to home for children with medical complexity and their families: an observational prospective cohort study protocol to assess the effectiveness of an innovative transitional care unit in the Netherlands (BRIDGE study)

**DOI:** 10.1136/bmjopen-2024-093693

**Published:** 2025-04-19

**Authors:** Heleen N Haspels, Misja C Mikkers, Hennie Knoester, Nicolaas J G Jansen, Inge M L Ahout, Jolanda M Maaskant, Liz van de Riet, Lotte Haverman, Karolijn Dulfer, Maartje Haasnoot, Mattijs W Alsem, Matthijs de Hoog, Job B M van Woensel, K F M Joosten, Clara D van Karnebeek, On behalf of the Transitional Care Unit Consortium, Bettina Sandbergen

**Affiliations:** 1 Department of Pediatric Intensive Care, Amsterdam UMC Locatie AMC, Amsterdam, The Netherlands; 2 Amsterdam Reproduction & Development Research Institute, Amsterdam UMC location University of Amsterdam, Amsterdam, the Netherlands; 3 Department of Neonatal & Pediatric Intensive Care, Division of Pediatric Intensive Care, Erasmus Medical Center – Sophia Children's Hospital, Rotterdam, The Netherlands; 4 Health Technology and Services Research Department, Technical Medical Centre, University of Twente, Enschede, The Netherlands; 5 Department of Pediatrics, University Medical Center Groningen, Beatrix Children’s Hospital, Groningen, The Netherlands; 6 Department of Pediatrics, Radboud University Medical Center, Amalia Children’s Hospital, Nijmegen, The Netherlands; 7 Department of Pediatrics, Amsterdam UMC location University of Amsterdam, Amsterdam, The Netherlands; 8 Child and Adolescent Psychiatry & Psychosocial Care, Emma Children's Hospital, Amsterdam UMC Location University of Amsterdam, Amsterdam, The Netherlands; 9 Department of Pediatrics, University Medical Centre Utrecht, Wilhelmina Children’s Hospital, Utrecht, The Netherlands; 10 Department of Rehabilitation, Physical Therapy Science and Sports, UMC Utrecht Brain Center, University Medical Center Utrecht, Utrecht, The Netherlands; 11 Departments of Pediatrics and Human Genetics, Emma Center for Personalized Medicine, Amsterdam UMC location University of Amsterdam, Amsterdam, The Netherlands

**Keywords:** Hospitals, Hospital to Home Transition, Organisation of health services

## Abstract

**Introduction:**

Children with Medical Complexities (CMC) often require 24/7 expert care, which frequently necessitates prolonged (re)admissions to a university medical centre (UMC), thereby impeding discharge to home. The transition from hospital to home for CMC is a multifaceted process with numerous challenges and obstacles. This protocol describes the evaluation of an innovative transitional care unit (TCU), where families can stay together in a home-like environment between hospital and home. Under the supervision of healthcare professionals, parents are supported in preparing for a sustainable home situation. We hypothesise that an intermediate stay at the Jeroen Pit Huis (JPH) will have a favourable effect on healthcare consumption, patient, parent and family-relevant outcomes in comparison to discharge directly from a hospital ward to home. Therefore, the purpose of this study is to compare the transition via the TCU JPH versus transition from hospital ward to home as provided elsewhere for CMC patients in the Netherlands.

**Methods and analysis:**

This observational prospective cohort study compares patients who transition directly from hospital to home with those who transition via the TCU. The control group comprises five UMCs in the Netherlands. Data will be collected by extracting information from electronic health records and through online questionnaires. Parents complete questionnaires at three time points: on discharge home, 3 months and 12 months postdischarge. Bayesian inverse probability weighting will be used to control for confounding effects and analyse the results.

**Ethics and dissemination:**

Ethical approval was granted by the Amsterdam UMC Medical Ethics Committee (W20_220#20.007). The need for ethical approval was waived by all other participating UMCs. Results will be disseminated through peer-reviewed scientific journals and conference presentations. The insights gained from this study will contribute to the development of a national care pathway to enhance transitional care for CMC and their families in the future.

**Trial registration number:**

NCT06599398 (ClinicalTrials.gov) - Bridging Hospital to Home for CMC and Their Families.

**STRENGTHS AND LIMITATIONS OF THIS STUDY**

- A prospective cohort design allows for the systematic comparison of children with medical complexity who are transferred home via the transitional care unit vs directly from hospital to home.
- Longitudinal follow-up over 12 months enables assessment of sustained intervention effects.
- The use of Bayesian Inverse Probability Weighting (BIPW) strengthens causal inference by adjusting for confounding.
- A limitation is that despite the use of BIPW, there may still be unmeasured confounding variables that could affect the results.
- High questionnaire burden may lead to selective participation, especially among highly stressed parents.

## Introduction

 Children with Medical Complexity (CMC) are characterised by one or more complex chronic conditions, technology dependency, high family needs and significant healthcare resource utilisation.^1^ The transition from hospital to home (H2H) for CMC is a multifaceted process with numerous challenges. The child’s primary caregivers, hereafter referred to as parents, transition from receiving care in the hospital to becoming the primary and most responsible caregivers at home. A study by van de Riet *et al*. revealed that parents experience and express needs across several domains—some of which remain unmet—including learning complex nursing care, gaining confidence, coordinating care, improving communication and access to information.^2^ Furthermore, families require support in accessing resources, finding support systems, navigating emotional challenges such as fatigue, fear, isolation and guilt, building effective relationships with professionals and adjusting their perspectives by establishing new routines.^2 3^

Due to these extensive needs, parents report feeling unsupported and unprepared during the transition to home.^4^,^7^ To address these challenges, various postdischarge interventions have been developed, including nurse visits and coaching,^8 9^ telephone follow-up calls^10^ and telemedicine.^11 12^ Other strategies for improving the H2H transition include the implementation of comprehensive care plans, complex care programmes and integrated delivery models.^13^ Comprehensive care plans facilitate better communication between parents and healthcare professionals,^14^,^16^ while complex care programmes reduce hospital-specific patient costs and also enhance parental satisfaction and family-centred care.^17^,^19^ In the Netherlands, an innovative transitional care unit (TCU), called the Jeroen Pit Huis (JPH), is developed to improve H2H care and address family needs.^20^

This TCU is situated on the premises of the Amsterdam University Medical Center (UMC) and intends to mimic the home situation. Families can stay together while practising in, and adapting to, their new reality until they are ready to transition home. The length of TCU stay varies from a minimum of 1 week to a maximum of 3 months. A multidisciplinary team of healthcare experts (nurses, psychosocial care workers, family counsellors and paediatricians) gradually guides parents towards taking on the role of their child’s primary caregiver. In this TCU, a structured care pathway is employed, addressing a critical gap in current healthcare systems that professionals recognise as essential.^21^,^23^ To date, the effectiveness of this novel care facility remains unknown.

The purpose of this study is to compare the transition via the TCU JPH versus transition from hospital ward to home as provided elsewhere for CMC patients in the Netherlands. We hypothesise that the continuous presence of parents in a home-like setting and the structured care pathway within the JPH will have a favourable effect on healthcare consumption, patient, parent and family-relevant outcomes, compared with discharge directly from a hospital ward to home.

## Methods and analysis

### Design

An observational prospective cohort study design was chosen, because it is not feasible to randomly assign patients to the intervention, as children are placed in a ward or the JPH based on the UMC where they are admitted. Random assignment to specific hospitals is not feasible given the practical impossibilities and negative impact of relocating children and families out of their own usual living environment.

The Strengthening the Reporting of Observational Studies in Epidemiology guideline will be used to report the study.^24^

### Setting

This study will be conducted in five Dutch UMCs and the TCU JPH, all part of the TCU consortium. The TCU consortium is a collaborative initiative between five UMCs, parents, patient associations and home care organisations.

The UMCs include the Amsterdam UMC – Emma Children’s Hospital, Erasmus MC (EMC) – Sophia Children’s Hospital, Radboudumc Nijmegen (Rumc) – Amalia Children’s Hospital, UMC Groningen (UMCG) – Beatrix Children’s Hospital and the UMC Utrecht (UMCU) – Wilhelmina Children’s Hospital.

### Intervention group

The JPH is a separate building on the premises of the Amsterdam UMC. This TCU consists of eight separate family apartments and was opened in May 2022. Each apartment has its own bedroom for the child with medical complexity, a master bedroom for parents or caregivers, a living room, a bathroom, a kitchenette, a mezzanine level with a bed and desk for (if necessary) siblings and a private outdoor area. Furthermore, the JPH has different communal areas: a kitchen, living room with various workspaces, relaxation and play, a snuggle room, an adapted bathroom for persons with disabilities, a physiotherapy gym and a garden with playground. The JPH primarily serves children from the Amsterdam UMC, although children can be referred from hospitals in or outside the Amsterdam region.

In the JPH, at least one parent must be present 24/7, and siblings are also welcome to stay in the apartment. The TCU team consists of nurses, family counsellors and paediatricians. Additionally, we collaborate with other healthcare professionals, including allied health professionals, home care providers and pharmacy personnel in both the hospital and home settings. Nursing care and supervision of a dedicated paediatrician is available 24/7. In this form (24/7 parental stay and nursing care under the supervision of paediatricians), the JPH is unique in the Netherlands. During the stay, more and more responsibilities and tasks are gradually transferred to the parents until they become the primary caregivers. This process is guided by a newly developed structured care pathway, which is informed by the parental needs identified within our consortium.^3^ This care pathway outlines the transition process for parents and children as they move from being care recipients to taking on the role of care providers and coordinating their own care. It is organised into seven phases, each with a step-by-step transition plan (see online supplemental file 1 for more details). Personalised treatment goals are established based on the specific needs of both the child and the parents, facilitating a successful transition back home. These needs will vary for each family but can be categorised under overarching domains of care, namely physical, financial, psychological, social, spiritual and practical aspects such as medical devices, legal considerations and future/developmental aspects.

### Control group

The control groups comprise patients and families who undergo the H2H transition directly from the hospital. This includes patients from the other four UMCs of the consortium. Patients from the Amsterdam UMC who transition directly from H2H without an intermediate stay in the JPH will also be included in the control group. Since there is no standardised care path, the H2H transitions vary across the different UMCs and even within different departments. There are no other TCUs in the Netherlands like the JPH; however, Erasmus MC offers one single-room apartment where parents can stay with their child for a weekend before being discharged home.^25^

A visual overview of differences between the intervention group and control group is presented in online supplemental file 1.

### Participants

**Figure 1:**
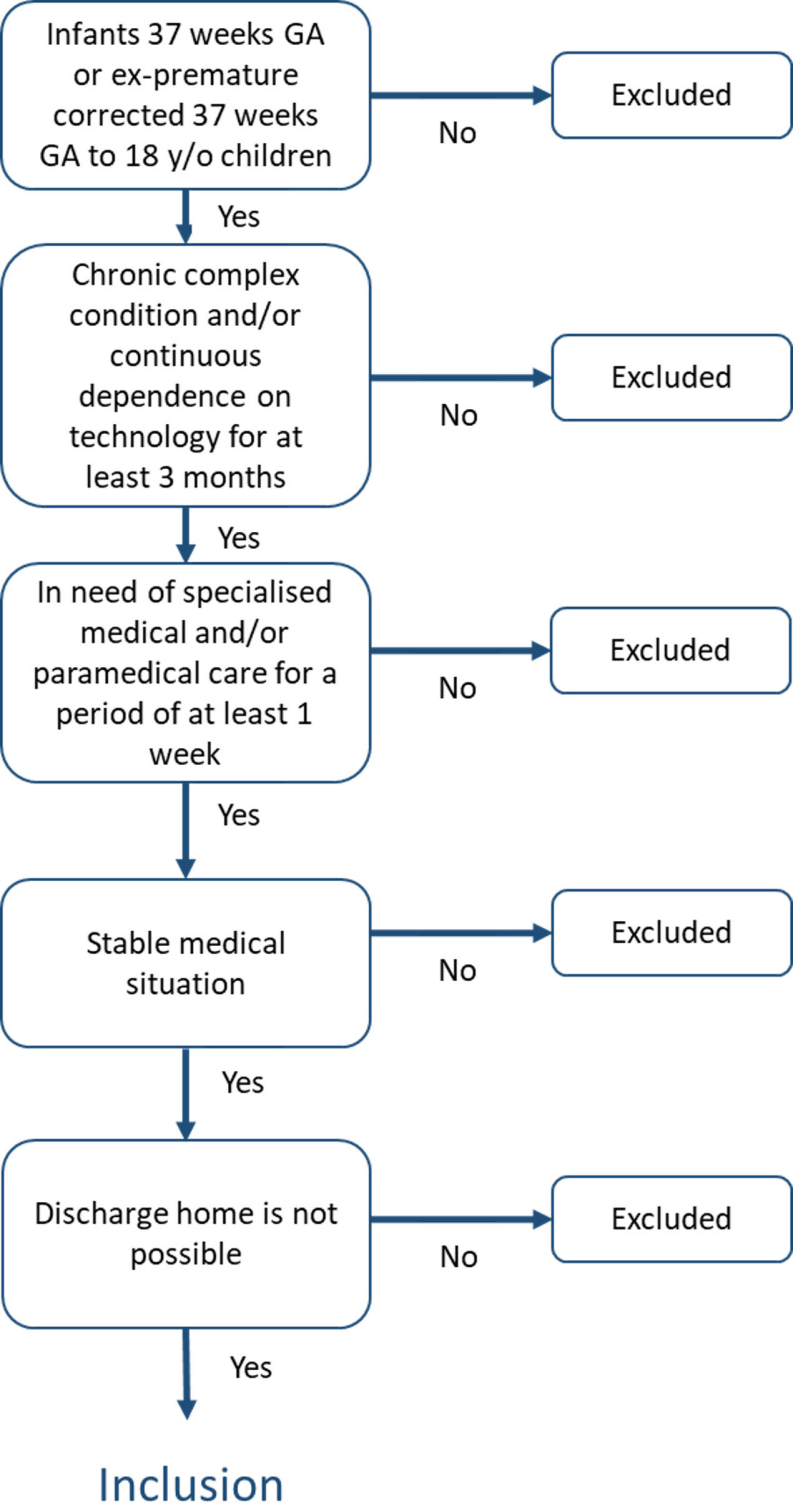
Flow chart of the inclusion process.

Potentially eligible participants are children admitted to one of the five UMCs. We will apply the following inclusion and exclusion criteria; a flowchart to further depict this is shown in figure 1.

#### Inclusion criteria

1. Age: at term (older than 37 weeks corrected gestational age) and younger than 18 years.
2. Admitted to the hospital with (a deterioration of) a chronic complex condition (CCC) and/or (expected) continuous dependence on technology after discharge (see online supplemental file 2 for detailed overview of CCC diagnosis).
3. Expected need for specialised medical and/or allied healthcare after discharge.
4. Discharge home not yet possible due to organisation circumstances, care-related or family circumstances (for more specifics see online supplemental file 3).
5. A stable medical condition and/or a set treatment regimen, defined as one or more of the following situations:
  1. A patent, safe airway to remain in the home situation, whether or not by means of a trachea cannula.
  2. Adequate respiration, whether or not by means of (intermittent) support with oxygen, non-invasive ventilation or invasive ventilation via a trachea cannula.
  3. Neurologically stable condition that may include temporary neurological impairments (such as seizures) not interfering (potentially life-threatening) with other vital functions such as respiration or circulation.
  4. Drug treatment that can be given at home where (if applicable): a nasogastric, duodenal or jejunal tube and/or a percutaneous endoscopic gastrostomy (PEG) tube or gastrostomy is in situ, or if necessary, a 'home-proof' intravenous access is guaranteed.
  5. In case of enteral tube feeding, the nasogastric, duodenal or jejunal tube and/or a PEG tube or gastrostomy is in situ, and the feeding schedule may be built up, reduced or variable if there are no contraindications for this. In case of parenteral nutrition, this must be organised according to the home situation.

#### Exclusion criteria

1. Patient requiring end-of-life care.
2. Existence of predominantly social/family issues without serious medical problems in the index patient.
3. Patient with behavioural/psychiatric problems necessitating other types of care.
4. Patient requiring inpatient rehabilitation medical care.
5. Patients discharged via other out-of-hospital facilities other than the JPH.

### Patient and public involvement

To ensure the inclusion of parents’ perspectives in the design of this study, close collaboration was maintained in the consortium with a parent caring for a CMC. She actively served as a representative of a patient advocacy organisation for children with complex medical needs (BS). She is engaged in research meetings and contributed to the development of the protocol. Furthermore, she took part in the pilot testing of the (self-constructed) questionnaires, provided feedback and, together with HH, made improvements to ensure feasibility. She provided insights and input for the parent and caregiver information flyer, letter and video clip.

### Outcomes

Outcomes were selected based on our previously developed Core Outcome Set (COS), established and published by our TCU consortium using a systematic review, the Delphi study, with healthcare professionals and focus groups with parents.^26 27^ A COS is considered a fundamental list of outcomes and is meant to add outcomes relevant to a specific study. Therefore, additional outcomes were selected through discussion within our TCU consortium. A full overview of outcomes including measurement tools and timing is presented in table 1.

**Table 1:** Overview outcomes, measurement tools/parameters and timing of outcomes BRIDGE study

	Outcome	Measurement tool/parameters	Measurement points
Primary outcome	Parental distress	Distress thermometer for parents	JPH/hospital discharge, 3 months and 12 months follow-up
Parent-focused (secondary) outcomes	Parental anxiety	PROMIS V.1.0 – anxiety 4a	JPH/Hospital discharge, 3 months and 12 months follow-up
	Parental depression	PROMIS V.1.0 – depression 4a	JPH/Hospital discharge, 3 months and 12 months follow-up
	Parental sleep disturbance	PROMIS Short Form V.1.0 sleep disturbance 4a	JPH/Hospital discharge, 3 months and 12 months follow-up
	Self-efficacy of parents	Parental measure of self-efficacy managing a child’s medications and treatments	JPH/Hospital discharge, 3 months and 12 months follow-up
	PTSD parents	PTSD checklist for Diagnostic and Statistical Manual of Mental Disorders-5	3 months and 12 months follow-up
	Financial impact	Self-constructed questionnaire (see online supplemental file 6)	12 months follow-up
Family-focused (secondary) outcomes	Impact on the life of the family	The family impact module of the Paediatric Quality of Life Inventory	JPH/hospital discharge, 3 months and 12 months follow-up
	Satisfaction with received care	Measures of processes of care	JPH/hospital discharge
Child-focused (secondary) outcomes	Child's quality of life	Standard set of patient reported outcomes and generic patient reported outcome measures for Dutch children (see online supplemental file 4)	JPH/hospital discharge, 3 months and 12 months follow-up
	Growth	Weight for age, weight for height and height for age	Study inclusion, JPH/hospital discharge, 3 months and 12 months follow-up
	Parent-perceived health status of the child	Self-constructed questionnaire (see online supplemental file 6)	JPH/hospital discharge, 3 months and 12 months follow-up
Health system (secondary) outcomes	Healthcare consumption	Length of hospital/JPH stay, number of (PICU) readmissions, emergency department visits and visits to the general practitioner (GP) or GP emergency services	12 months of follow-up

JPH, Jeroen Pit Huis ; PICU, pediatrics intensive care unit; PTSD, post-traumatic stress disorder.

#### Primary outcome

The primary outcome is parental distress at discharge home and will be measured by the distress thermometer for parents (DT-P).^28^ This includes (1) a thermometer score regarding overall distress in the past week, with a score from 0 (no distress) to 10 (extreme distress), (2) a problem list which inquired the occurrence of 37 (child age <2 years) or 34 (child age≥2 years) everyday problems over the past week across six domains (practical, family/social, emotional, physical, cognitive and parenting), where problem domain scores are the sum of item scores (yes=1, no=0) within that problem domain and (3) additional questions concerning: perceived support from surroundings, perceived lack of understanding from people concerning their situation and parental chronic illness. Internal consistency of the DT-P was in the range of (*α*) = 0.52–0.89.^29^

#### Parent-focused (secondary) outcomes

The measured outcomes for parents will include anxiety, depressive symptoms, sleep disturbance (SD), self-efficacy, post-traumatic stress disorder (PTSD) and financial impact.

The PROMIS V.1.0 – anxiety 4a and the PROMIS V.1.0 – depression 4a short forms will be used to assess symptoms of respectively anxiety and depression in parents.^30^ Both measures comprise 4 items, scored on a 5-point Likert scale ranging from ‘never’ to ‘almost’ using a 7-day recall period. Total raw scores are calculated and transformed into a T-score with a mean of 50 and SD of 10. Higher scores indicate more anxiety or depression symptoms. Psychometric properties of Patient Reported Outcome Measurements (PROMS) are sufficient.^31^

Parental SD will be assessed with the short form 4a PROMIS SD.^32^ The 4-item questionnaire will be used to measure self-reported perceptions of sleep quality, depth and restoration within the past 7 days. This measure comprises a 5-point Likert scale ranging from ‘never’ to ‘almost’ using a 7-day recall period. The raw score will be converted into a T-score with a mean of 50 and SD of 10. Higher scores indicate more sleep/wake disturbances. Psychometric properties of PROMS are sufficient.^31^

Self-efficacy will be measured using the Parental Measure of Self-Efficacy Managing a Child’s Medications and Treatments.^33^ This tool is adapted from the PROMIS self-efficacy patient-reported outcome (PRO) measure that assesses adults’ self-efficacy in managing their own medications and treatments.^34^ The scale consists of 30 items, of which 14 items are relevant to children regardless of condition severity (eg, healthcare information/decision making; symptom identification/management) and 16 items are relevant to children with specific healthcare needs (eg, medication usage, equipment). This measure comprises a 5-point Likert scale ranging from ‘I am not confident at all’ to ‘I am very confident’. Scores will be obtained by summing the mean scores of the 30 items, ranging between 30 and 150, with higher scores indicating higher self-efficacy. The questionnaire, initially available only in English, underwent translation into Dutch using the Functional Assessment of Chronic Illness Therapy translation methodology, a method recommended by the PROMIS Health Organisation.^35 36^ For an overview of the translation process, see online supplemental file 5. No psychometric properties are available yet.

PTSD checklist (PCL) for Diagnostic and Statistical Manual of Mental Disorders (DSM)-5 (PCL-5) will be used to screen for symptoms of PTSD, with a 1-month recall period, based on the DSM-IV criteria. The PCL-5 includes 20 items, scored on a Likert scale ranging from 0 to 4, reflecting the DSM-5 diagnostic criteria of PTSD. A higher sum score represents more pronounced PTSD symptoms, and a cut-off score of 31 was used to screen for PTSD. Four subscales can be calculated: intrusion, avoidance, emotion alterations and hyperarousal.^37^ Internal consistency of the PCL-5 is (*α*) = 0.93.^38^

Financial impact on parents will be measured through a self-conducted survey, developed by the authors in consultation with TCU consortium members, including a parent representative. This survey will assess the financial impact based on the ability to work and changes in the financial situation due to the consequences of the child’s illness (see online supplemental file 6).

#### Family-focused (secondary) outcomes

The assessed outcomes for families will encompass the impact on their overall family life and satisfaction with the care received, emphasising the extent to which these services are family-centred.

The Pediatric Quality of Life Inventory Family Impact Module (PedsQL FIM) will be used to assess family impact in the last 4 weeks.^39^ The PedsQL FIM measures parent self-reported physical, emotional, social and cognitive functioning, communication and worry. Additionally, parent-reported family daily activities and family relationships will be measured. The scale consists of 36 items with a 5-point response scale (0=never a problem; 4=always a problem). The questionnaire yields three summary scores: the parent health-related quality of life summary score (which is calculated by averaging items in the physical, emotional, social and cognitive functioning scales), the family functioning summary score (which is calculated by averaging items in the daily activities and family relationships scales) and the total impact score (which is calculated by averaging items in all eight scales). Internal consistency of the English PedsQL FIM was in the range of (*α*) = 0.82–0.97.^39^ No Dutch psychometric properties are available yet.

Parental perceptions of care will be evaluated using the Measures of Processes of Care (MPOC-20),^40 41^ which measures caregiver perceptions of the extent of family-centredness of the care received by children with disabilities in a healthcare organisation. The MPOC-20 is composed of 20 items covering five scales (1) enabling and partnership; (2) coordinated and comprehensive care; (3) providing specific information about the child; (4) respectful and supportive care; and (5) providing general information. The items are answered on a 7-point Likert scale ranging from 1 (not at all) to 7 (to a very great extent). A higher score reflects a more favourable judgement of the care process. Internal consistency of the MPOC-20 was in the range of (*α*) = 0.78–0.91^42^

#### Child-focused (secondary) outcomes

The assessed outcomes for CMC will encompass the child’s quality of life, the parent-perceived health status of the child and growth.

Overall quality of life scores and physical, social and mental functioning and fatigue and pain symptoms of the child will be measured according to the standard set of PROs and generic PROMS for Dutch children.^43^ Depending on the age of the children, different questionnaires need to be completed proxy or self-reported (online supplemental file 4). Psychometric properties of PROMS are sufficient^44^,^47^

One question, formulated by the research group, aims to assess the parent-perceived health status of the child. Parents are prompted to indicate their child’s current health condition on a scale from 0 to 100, where 100 represents the best health condition imaginable, and 0 represents the worst health condition imaginable (online supplemental file 6).

Data on standard anthropometric measurements (weight and height) will be collected (if present) at study inclusion, on discharge from the hospital/JPH and 3 months and 12 months after discharge. This data will be extracted from the electronic health record. Interpretation of weight and height will be performed by means of calculating z-scores according to age: weight for age for children <1 year old and weight for height for children older than 1 year old. Height for age will be calculated for all children.

#### Health system (secondary) outcomes

Healthcare consumption data will be extracted from the electronic health records, including: the length of hospital/JPH stay, the number of (pediatric intensive care unit) readmissions, emergency department visits and visits to the general practitioner (GP) or GP emergency services in the year following discharge.

### Recruitment

Parents will be recruited during hospitalisation in one of the participating UMCs and/or stay in the JPH. The study will be advertised to clinicians in relevant departments with a request to consider whether patients on the ward may be eligible. Screening of patients will be done weekly. The treating physician will ask eligible candidates for permission to be contacted by the researcher. The local researcher will then explain the study orally and in writing to the parents and provide a patient information letter. If interested, parents provide online informed consent. Children aged 12–18 must also provide their own online consent. For all patients, it is checked whether they are already enrolled in a standard follow-up programme to ensure that questionnaires are not duplicated. This is done only after obtaining informed consent from the participants.

### Data collection and management

On obtaining written informed consent, the researcher will assign a research subject ID number to patients. Every UMC has its own encryption key. Medical and healthcare consumption data will be extracted by the research team from the electronic health records and will be entered in a password-protected online database (Castor EDC). Parent reported data will be collected through online questionnaires using the KLIK PROM portal, which is an online portal to systematically monitor PROs in children with various chronic diseases and their parents over time.^48^

A KLIK research website is specifically created for the BRIDGE study, providing access to study information and allowing completion of questionnaires. The website is accessible only to the study team with a password (www.bridgetrial.nl). Parents receive a unique login token, allowing them to complete the questionnaires online, with the flexibility to take breaks as needed. The expected required time-investment of the parents to fill in the questionnaire is approximately 2.5 hours in total (around 50 min at three different time points).

The questionnaire completion windows are defined as follows:

- On hospital discharge, participants are expected to fill out the questionnaire within the period of 48 hours before and 48 hours after discharge.
- At the 3-month follow-up, the window extends to 2 weeks before and 2 weeks after this point.
- At the 12-month follow-up, the window extends by 2 weeks before and 2 weeks after this point.

Parents will receive a one-time €20 gift card as a token of appreciation for their participation.

### Statistical analysis

Analysis of the primary and secondary outcomes will be done using Bayesian inverse probability weighting methods.^49^,^51^ All analyses will be performed using RStudio software (V.4.0.3). In online supplemental file 7 and an online blog, we demonstrate through data simulation how we will measure the effect, including the pertinent R code and rationale behind our methodological selection.

### Bayesian analysis

Bayesian analysis principles will be used to evaluate the effect of the JPH. This approach is adopted due to the constrained data points available in the BRIDGE study. Unlike traditional significance tests, which focus on determining whether an effect is statistically significant by testing how likely the observed data are conditional on the null hypothesis being true, our Bayesian analysis aims to estimate the probability that the intervention has a specific effect given the data. Bayesian methods are particularly beneficial for relatively small sample sizes, because they allow the incorporation of prior information and provide more intuitive and interpretable results.^49^ Additionally, Bayesian analysis can produce credible intervals that directly reflect the uncertainty about parameter estimates, making it a robust choice for our study with limited data.

### Bayesian inverse probability weighting

**Figure 2:**
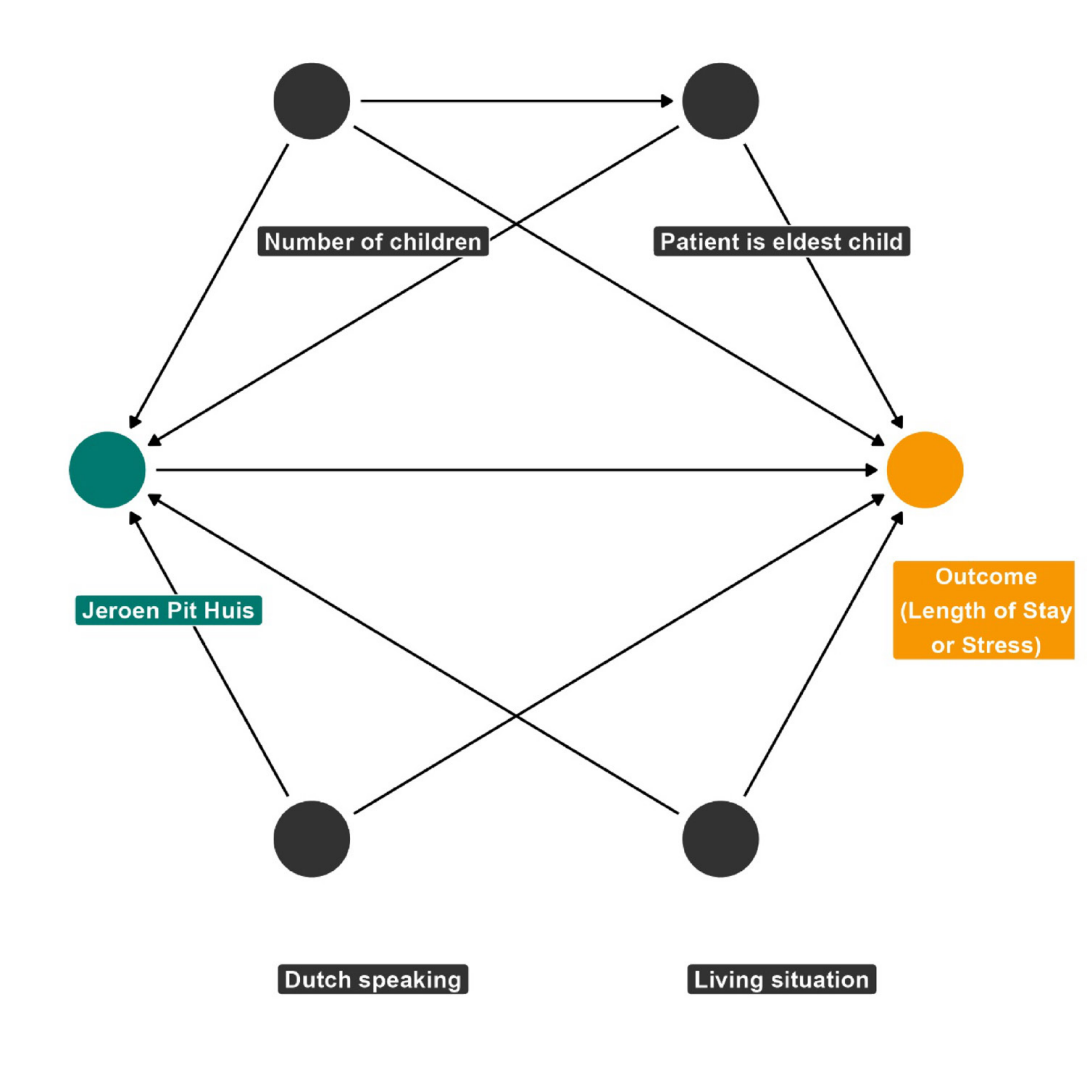
Simplified generic directed acyclic graph for hospital length of stay and parental stress.

The intervention and control groups differ in characteristics and outcomes, and without randomisation, these differences can confound the results. The Directed Acyclic Graph (DAG) (figure 2) visually represents the relationships between the variables and helps to identify the confounders that need to be adjusted for in the analysis. A simplified generic DAG has been used initially; however, a specific DAG will be developed for each outcome. The DAG clarifies the causal pathways and ensures that we appropriately control for variables that could bias the results. To control for confounding effects, we use Bayesian inverse probability weighting, following these steps:

A model will be created to predict treatment. Confounders identified with the DAG will be used as covariates to predict the probability of each patient being in the treatment group using a logistic regression (Bernoulli distribution), generating ‘propensity scores’. The regression model will be:


$$Treatment\;=\;\beta_{0}\;+\;\beta_{1}X^{T}+\varepsilon$$

where, $X^{T}$ is the matrix of confounders.

Samples of propensity scores will be generated: many propensity scores will be obtained from the posterior distribution.

Propensity scores will be converted into inverse probability of treatment weights using the formula:


$$\frac{\text{Treatment}}{\text{Propensity score}}+\frac{1-\text{Treatment}}{1-\text{Propensity score}}$$

An outcome model will be run using these weights: a Gaussian model will be used for numerical outcomes, and a cumulative probit model will be used for ordinal outcomes. The regression model used for the outcome analysis will be:


$$Outcome\;=\;\gamma_{0}\;+\;\gamma_{1}\times Treatment\;+\;\varepsilon$$

### Additional analyses

Baseline patient characteristics and descriptive variables will be presented for the intervention and control group: age, gestational age, sex, diagnosis, underlying chronic condition, reason for admission, age patient during onset disease, medications after discharge, medical devices after discharge and amount of home care, medical day care or respite care. Parent characteristics include: age, gender, country of birth, ethnicity, Dutch speaking, marital state, educational and occupational level. Family characteristics include: number of children in the family, age of siblings, position of the index child within the family. To describe patient, parent and family characteristics, numerical data will be summarised using descriptive statistics: mean and SD for normally distributed data, median and quartiles for non-normally distributed data.

### Sample size

To assess the largest possible sample size within the available resources, the original plan involved enrolling all eligible patients over a span of 1.5 years, with the aim of accruing approximately 150 patients (50 in the intervention group and 100 in the control group).

The numbers are based on our previous pilot study: H2H transition of CMC in the Netherlands: current practice.^52^ In this study, the same population was included and the objectives were to (1) describe the demographics, clinical characteristics and course of patients who are in the H2H transition and (2) identify perceived barriers for postponement of H2H transition (METC Amsterdam UMC reference number W21_581 # 22.025). This study enrolled 44 patients over a period of 6 months at four UMCs (Amsterdam UMC, EMC, UMCG, Radboud UMC). In the BRIDGE study, parents are asked to fill in questionnaires, which may result in fewer parents willing to participate. However, to compensate for this, an additional UMCU has been included.

### Study limitations

This study protocol faces several inherent limitations. First, the substantial time commitment for parents, approximately 50 min at discharge home, may discourage those with high stress levels from participating. This could result in an under-representation of highly stressed parents, particularly in the control groups, given the hypothesis that parents in the JPH group experience less stress. Second, the exclusion of families with limited proficiency in both English and Dutch may result in a skewed representation of the population. These excluded families, often facing distinct challenges in the transition from H2H, represent a potential gap in our understanding of their H2H experience. Third, the decision to exclude patients who are transferred to another out-of-hospital facility might have the consequence that the different groups are less comparable because the included control group might be less care-intensive (because they can be discharged home directly instead of having to stay in an out-of-hospital facility for a period). Finally, a limitation is that despite the use of Bayesian inverse probability weighting, there may still be unmeasured confounding variables that could affect the results. The impossibility of randomly selecting the intervention, of course, is the biggest issue to this matter. A careful selection of control patients may help decrease the chances of bias.

### Trial status

Currently, all five UMCs are enrolling patients. Recruitment began in December 2023 at the JPH and Amsterdam UMC, followed by UMCG in January 2024, Radboud in March 2024, EMC in April 2024 and UMCU in September 2024. Final inclusion of the BRIDGE study is expected in 2026.

## Supplementary material

10.1136/bmjopen-2024-093693 online supplemental file 1

10.1136/bmjopen-2024-093693 online supplemental file 2
