# Supplementary material for: Bridging hospital to home for children with medical complexity and their families: an observational prospective cohort study protocol to assess the effectiveness of an innovative transitional care unit in the Netherlands (BRIDGE study)
Source: BMJ Open. 2025 Apr 19;15(4):e093693. doi: 10.1136/bmjopen-2024-093693 (PMC12010278; doi:10.1136/bmjopen-2024-093693)
Supplement: online supplemental file 1 [file bmjopen-15-4-s001.docx]

# Supplement 1: JPH care vs usual care


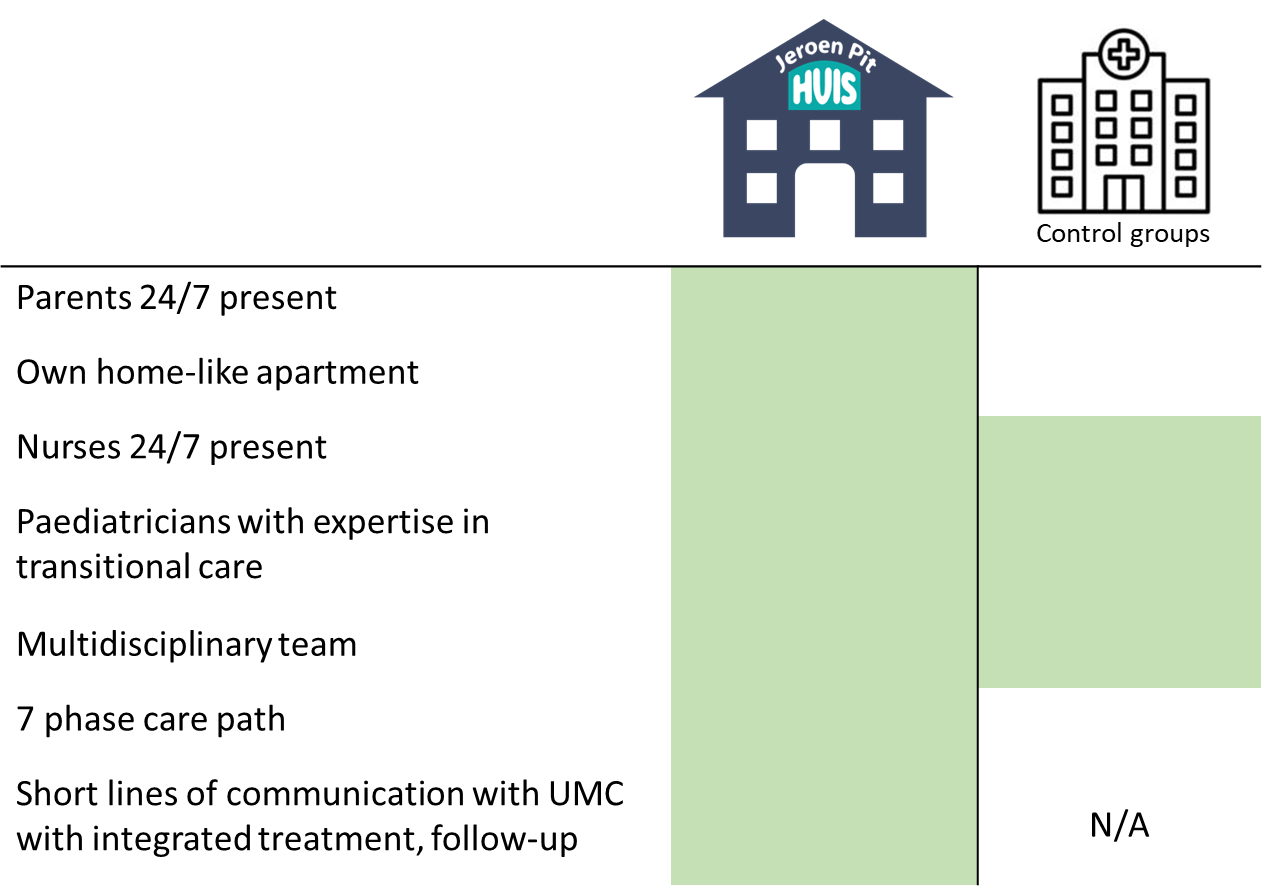


Figure 1 characteristics intervention and control groups. Abbreviations: N/A: not applicable

Below is an overview of the seven phase care path.

Hospital Phase

- Step 1: Screening Patients
  - The hospital team initiates early discharge planning, identifies potential TCU candidates, and consults the TCU pediatrician or nurse.
  - Standardized screening ensures a comprehensive initial care plan before transitioning to the TCU.

Transitional Care Unit (TCU) Phase

- Step 2: Adjustment at TCU
  - Families acclimate to the new setting and recover from hospital-related stress.
  - Transition goals are refined, and a final care plan is developed.
- Step 3: Training at TCU
  - Parents balance caregiving responsibilities with their own well-being.
  - They work on transition goals, adjust care when necessary, and build confidence in managing their child’s care and decision-making.
- Step 4: Finalizing at TCU
  - Preparing for discharge by finalizing remaining aspects of care.
  - Ensuring home care arrangements and establishing the primary care team responsible for post-discharge support.
  - Transitioning from the TCU to home.

Home Phase

- Step 5: Discharge Home
  - Parents assume full responsibility for their child’s care in a safe home environment.
  - Necessary care and support services are in place, and parents know where to seek help if needed.
- Step 6: Stable at Home
  - The family regains a sense of normalcy and control over their child’s care.
  - Parents manage caregiving independently but maintain access to necessary support networks.
  - They can easily reach primary care providers and other relevant professionals when needed.
- Step 7: Step-Up Function
  - If necessary, families can receive additional support, including planned or unplanned hospital readmission.
  - This ensures a safety net for families facing unforeseen challenges.

# Supplement 2: Chronic Complex Condition (CCC)

A complex chronic condition (CCC) is defined according to Feudtner et al. (1) as “any medical condition that can be reasonably expected to last at least 12 months (unless death intervenes) and to involve either several different organ systems or one organ system severely enough to require specialty pediatric care and probably some period of hospitalization in a tertiary care center.” Verlaat et al., updated the CCC list based on expert opinion (2). See table 1 for diagnosis classified as CCC. An unknown but suspected complex and chronic condition, such as a child born with multiple congenital anomalies but lacking a unifying diagnosis, will be included.

| **Complex chronic conditions (CCCs)** | |
| --- | --- |
| **Subgroup** | **Diagnoses from the PICE database (ANZPIC diagnose list)** |
| **Cardiovascular** | Absent pulmonary valve syndrome* Anomaly of the coronary artery Arterial switch* Atrioventricular septal defect Cardiomyopathy Cavo pulmonary shunt* Cor triatriatum Double outlet right ventricle Ebstein’s anomaly Fontan procedure* Hypoplastic left heart syndrome Hypoplastic left ventricle* Hypoplastic or interrupted aortic arch* Hypoplastic right ventricle* Levo transposition of the great arteries Mitral valve stenosis  Monoventricle Norwood procedure – step 1* Pacemaker insertion/revision* Portal hypertension* Pulmonary atresia or stenosis Pulmonary artery banding* Reconstruction of aortic arch* Reconstruction of left ventricular outflow* Reconstruction of right ventricular outflow* Restoration of atrioventricular septal defect* Repair of plastic pulmonary artery* Repair or replacement of conduit* Repair of tetralogy of Fallot* Right ventricular outflow tract obstruction* Senning procedure* Supraventricular arrhythmia Surgery of pulmonary collateral arteries* Systemic to pulmonary shunt procedure* Tetralogy of Fallot Total abnormal pulmonary venous return Transplantation of heart Transplantation of heart and lung Transplantation of heart and lung – state after procedure Transposition of the great arteries Tricuspid atresia or stenosis Truncus arteriosus Vasculitis* Ventricular arrhythmia |
| **Respiratory** | Bronchiectasis  Central apnea* Choanal atresia or stenosis* Chronic lung disease* Congenital lung disease Cystic fibrosis Infant respiratory distress syndrome* Laryngomalacia Malacia trachea or bronchus Mediastinal mass* Pulmonary edema Pulmonary hypoplasia Pulmonary insufficiency* Reconstruction of larynx* Subglottic stenosis Tracheostomy* Trachea or bronchus stenosis Transplantation of lung Transplantation of lung – state after procedure Vocal cord paralysis* |
| **Hematological** | Coagulation defects Hematologic disease* |
| **Endocrinological** | Congenital metabolism disorder Diabetes (comorbidity)* Diabetes insipidus Diabetes mellitus with ketoacidosis Diabetes mellitus without ketoacidosis Endocrine disorder Kasaï procedure* |
| **Gastrointestinal** | Biliary atresia Colitis Congenital diaphragmatic hernia Gastroschisis or exomphalos Hirschsprung’s disease* Liver disease – other* Esophageal atresia Repair of esophageal atresia* Repair of esophageal fistula* Repair of total anomalous pulmonary venous return* Short bowel syndrome* Transplantation of kidney Transplantation of liver Transplantation of liver – state after procedure Transplantation of small intestine Varices of esophagus or stomach* |
| **Immunological** | Congenital immunodeficiency Graft versus host disease Neutropenia* Pancytopenia* Pheochromocytoma* |
| **Neuromuscular** | Acute disseminated encephalomyelitis* Arnold-Chiari malformation Brain arteriovenous malformation* Brain tumor Central nervous system shunt dysfunction or infection* Cerebral aneurysm Cerebral cyst Cerebral infarction* Chronic traumatic encephalopathy  Congenital brain disease* Convulsions* Craniotomy – fossa anterior* Epilepsy (comorbidity) Hydrocephalus Insertion of revision of central nervous system shunt* Lobectomy or hemispherectomy* Meningomyelocele or spina bifida  Muscular dystrophy Myasthenia gravis Myelum – impairment* Myopathy Repair of myelomeningocele* Static encephalopathy |
| **Oncological** | Cystic hygroma Leukemia or lymphoma Malignant solid organ neoplasm Transplantation of bone marrow Transplantation of bone marrow – state after procedure |
| **Renal** | Chronic kidney failure Hydronephrosis* Nephrotic or nephritic syndrome* Transplantation of kidney – state after procedure |
| **Endocrinal** | Syndrome of inappropriate antidiuretic hormone secretion* |
| **Genetic** | Chromosomal abnormality Craniosynostosis* DiGeorge syndrome Down syndrome Pierre Robin syndrome* |
| **Urological** | Repair of exstrophia vesicae* |
| **Miscellaneous** | Syndrome or malformation* |
| * Diagnoses that were not on the original list (as CCC) | |

# Supplement 3: Reasons discharge home is not possible

Organization-, care- or family circumstances include the lack of 1 or more of the following requirements for safe discharge home:

1. A sustainable care plan using the four ‘Medische Kindzorg Systeem’ domains (medical, safety, development and social) has been drawn up that includes all four domains such as among others (Medical Child care System that is used in The Netherlands to structure medical child care in the home situation):

- care needs
- clear coordination of tasks and responsibilities of parents and healthcare professionals (primary, secondary and tertiary care).

1. The necessary medical and nursing care support (such as a feeding pump, adequate home care).
2. Parents know who to contact with questions about the organization of care (e.g. financial issues, wheelchair, municipality)
3. Essential medical technology is available at home and can be used for care.
4. Reimbursement of care at home is adequately arranged.
5. The house is located and furnished in such a way that the child can stay in it safely (accessibility for emergency services, telephone contact with parents, technical facilities

Parents/informal carers are adequately trained to take care of the child in the home situation, that means that they:

1. Master care in all areas (medical, nursing, technical, psychosocial)
2. Obtain declaration of care competencies, in particular:

- assessment for somatic deterioration
- acting in emergency situations (e.g. seeking help, resuscitation and if applicable: cannula dislocation, PEG probe problems, docking of a seizure)
- realizing when to call for help and whom to call for help

1. Can obtain adequate telephone assistance (working telephone, English/Dutch speaking)
2. Are psychologically and emotionally ready to deal with new home situation (according to parent and care professionals)

# Supplement 4: Standard set of patient reported outcomes (PROs) and generic patient reported outcome measures (PROMs) for Dutch children

|  | **Children 0 - 4 years old (Parent Report Measures)** | **Children 5 - 7 years old (Parent Report Measures)** | **Children 8 - 17 years old (Child Report Measures)** |
| --- | --- | --- | --- |
| **Well-being/quality of life** | NRS Well-being/quality of life Parent Proxy | NRS Well-being/quality of life Parent Proxy | NRS Well-being/quality of life |
| **Perceived health** | NRS Perceived Health Parent Proxy | NRS Perceived Health Parent Proxy | NRS Perceived Health |
| **Social functioning / participation** | not available | PROMIS Parent Proxy Relation with Peers – Short Form 4a | PROMIS Pediatric Relation with Peers – Short Form 4a |
| **Physical functioning** | not available | PROMIS Parent Proxy Mobility – Short Form 4a | PROMIS Pediatric Mobility – Short Form 4a |
| **Anxiety** | PROMIS Early Childhood Anxiety - Short Form 4a | PROMIS Parent Proxy Anxiety - Short Form 4b | PROMIS Pediatric Anxiety - Short Form 4b |
| **Depression** | PROMIS Early Childhood Depressive Symptoms - Short Form 4a | PROMIS Parent Proxy Depressive Symptoms - Short Form 4b | PROMIS Pediatric Depressive Symptoms - Short Form 4b |
| **Anger** | PROMIS Early Childhood Anger Scale - Short Form 4a | PROMIS Parent Proxy Anger Scale - Short Form 5a | PROMIS Pediatric Anger Scale - Short Form 5a |
| **Fatigue** | NRS Fatigue Parent Proxy | NRS Fatigue Parent Proxy | NRS Fatigue |
| **Pain** | NRS Pain Parent Proxy | NRS Pain Parent Proxy | NRS Pain |
| **Sleep** | NRS Sleep Parent Proxy | NRS Sleep Parent Proxy | NRS Sleep |
| Abbreviations: NRS; Numeric Rating Scale, PROMIS; Patient-Reported Outcomes Measurement Information System | | | |

# Supplement 5: Translation process Parental Measure of Self-Efficacy Managing a Child’s Medications and Treatments

The translation was performed according to the FACIT translation methodology. This methodology was developed to establish equivalence of meaning and measurement between the different versions of the questionnaire targeted to different languages (8). Steps 1–8 from this methodology were performed in this study to achieve linguistic, content, and conceptual equivalence.

- **Step 1:** Forward translation. Two forward translations of the source items were performed, both native Dutch speakers highly competent in both languages, where one has a medical background (A.M.), and one is a medical doctor (S.B.). Translators were instructed to translate the source items into simple and logical Dutch sentences.
- **Step 2:** Reconciliation. The two forward translations were reconciled by a native Dutch speaker who is a medical doctor (M.H.), in order to achieve equivalence.
- **Step 3:** Back translation. The reconciled Dutch version of the Parental Measure of Self-Efficacy Managing a Child’s Medications and Treatments was then translated back into English by a professional translator with an high expertise in translation methodology (P.H.). The back-translator was blinded to the original source English version.
- **Step 4:** Quality control: compare back translations to source. The backward-translated versions were compared to the source items of the Parental Measure of Self-Efficacy Managing a Child’s Medications and Treatments questionnaire by the main author (H.N.H.) to identify discrepancies. In case of evident differences between the source item and the backward translation, the forward translations were reviewed, and if necessary, the translated items were adapted to match the source item.
- **Step 5:** Independent review. A native Dutch speaker, with an expertise in PROMs (M.M.), examined all the preceding steps. In this step, previous Dutch translations of the PROMIS Self-efficacy item bank was also considered as one of the possible translations. Existing ambiguity in the translations was discussed and items were adapted if necessary.
- **Step 6:** Harmonization, quality control, and proofreading. A proofreader, who was not involved in the earlier translation process, was asked to assess the translation process and take a critical look at the spelling and sentence structure (I.Z.). The first author implemented the improvement suggestions and finalized the questions for cognitive debriefing.
- **Step 7:** Cognitive debriefing. A stratified group of five Dutch parents with a chronically ill child were asked to participate in a cognitive debriefing to assess the comprehensibility of the translated items. The interviews were performed by the main author, who was trained to conduct interviews. All interviews were audio-recorded. The audio recordings were analyzed, and comments about whether the adaptation of an item was necessary were reviewed.
- **Step 8:** Finalization of final version. IZ an HH reviewed all prior steps and finalized the questionnaire including formatting, typesetting, and proofreading.


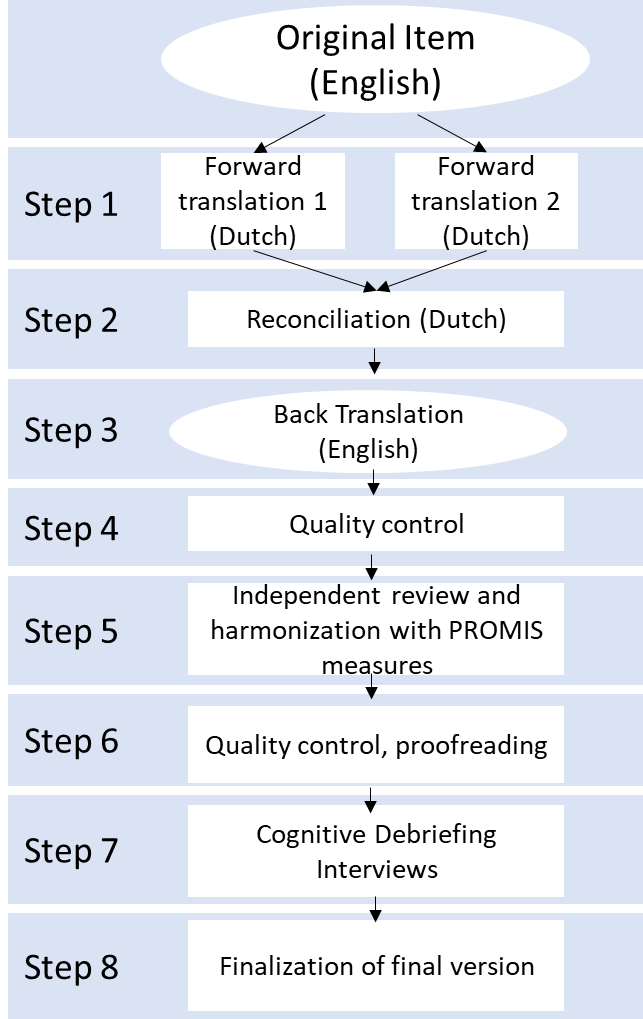


Figure 2 Schematic overview translation process

# Supplement 6: Self-constructed questionnaires

*Parent-perceived health status of the child questionnaire*


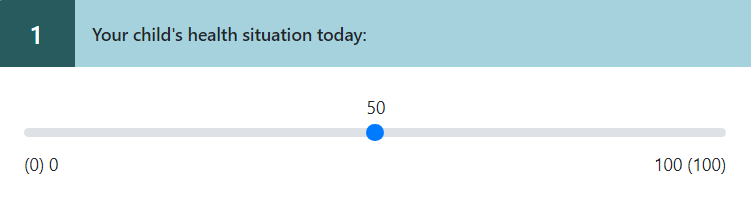
We would like to ask you to indicate on the scale below how good or how bad you think your child’s health is today. On this scale, “100” means the best health condition imaginable, and “0” means the worst health condition imaginable. Move the slider to the point on the scale that you think indicates how good or bad your child’s health is today.

*Financial impact questionnaire*

Question 1

What is your current work situation? (more than one answer possible)

- Paid work (Go to Q1a + b)
- Housewife/househusband
- Unemployed/job seeking
- Disabled (WAO, AAW)*
- Student
- Full-time caregiver
- Other, namely___

* The Netherlands follows the Disability Insurance Act (WAO) for disability benefits, with the General Disability Act (AAW) having been replaced by WAO on January 1, 1998.

Question 1a

How many hours do you work per week? (open question)

Question 1b

What is your occupation at the moment? (open question)

Question 2

Did you modify your work situation as a result of your child’s illness?

- No, not modified
- Yes, I have reduced my working hours, namely ­­­___ hours less per week
- Yes, I have increased my working hours, namely ­­­­___ hours more per week
- Yes, I have changed my job, with the reason ___
- Yes, other namely___

Question 3

Has the financial situation changed as a result of your child's illness?

- Nothing has changed
- Our financial situation has deteriorated, but we can get by
- Other, namely___

If two parents are responsible for the child, both are required to fill out these questions.

# Supplement 7: Bayesian inverse probability weighting

# Datasimulation for BRIDGE study using bayesian inverse probability weighting methods -> other file is separately attached

# References supplementary

1. Feudtner C, Feinstein JA, Zhong W, Hall M, Dai D. Pediatric complex chronic conditions classification system version 2: updated for ICD-10 and complex medical technology dependence and transplantation. BMC pediatrics. 2014;14(1):1-7.

2. Verlaat CW, Visser IH, Wubben N, Hazelzet JA, Lemson J, Van Waardenburg D, et al. Factors associated with mortality in low-risk pediatric critical care patients in the Netherlands. Pediatric critical care medicine. 2017;18(4):e155-e61.

3. van Oers HA, Schepers SA, Grootenhuis MA, Haverman L. Dutch normative data and psychometric properties for the Distress Thermometer for Parents. Quality of Life Research. 2017;26:177-82.

4. Foster CC, Blackwell CK, Kan K, Morales L, Cella D, Shaunfield S. Parental self-efficacy managing a child’s medications and treatments: adaptation of a PROMIS measure. Journal of patient-reported outcomes. 2023;7(1):1-12.

5. Van Praag DL, Fardzadeh HE, Covic A, Maas AI, von Steinbüchel N. Preliminary validation of the Dutch version of the Posttraumatic stress disorder checklist for DSM-5 (PCL-5) after traumatic brain injury in a civilian population. PloS one. 2020;15(4):e0231857.

6. Varni JW, Sherman SA, Burwinkle TM, Dickinson PE, Dixon P. The PedsQL™ family impact module: preliminary reliability and validity. Health and quality of life outcomes. 2004;2(1):1-6.

7. Siebes RC, Maassen GH, Wijnroks L, Ketelaar M, van Schie PE, Gorter JW, Vermeer A. Quality of paediatric rehabilitation from the parent perspective: validation of the short Measure of Processes of Care (MPOC-20) in the Netherlands. Clinical Rehabilitation. 2007;21(1):62-72.

8. Eremenco SL, Cella D, Arnold BJ. A comprehensive method for the translation and cross-cultural validation of health status questionnaires. Evaluation & the health professions. 2005;28(2):212-32.
