# Supplementary material for: Bridging hospital to home for children with medical complexity and their families: an observational prospective cohort study protocol to assess the effectiveness of an innovative transitional care unit in the Netherlands (BRIDGE study)
Source: BMJ Open. 2025 Apr 19;15(4):e093693. doi: 10.1136/bmjopen-2024-093693 (PMC12010278; doi:10.1136/bmjopen-2024-093693)
Supplement: online supplemental file 2 [file bmjopen-15-4-s002.pdf]

Evaluation of a pediatric Transitional Care Unit in the Netherlands

Datasimulation for BRIDGE study using bayesian inverse probability weighting methods

INNOVATIVE CARE MODEL | BAYESIAN PROBABILITY WEIGHTING METHODS | DATASIMULATION

In this post, we demonstrate through data simulation how we will measure the effect of an innovative Transitional Care Unit (TCU) called the Jeroen Pit Huis (JPH). We are using bayesian probability weighting methods.

AUTHORS | AFFILIATIONS

Heleen Haspels — Amsterdam UMC location University of Amsterdam, Emma Children's Hospital & Erasmus Medical Center - Sophia Childrens hospital

Misja Mikkers — University of Twente

PUBLISHED

March 20, 2024

1 Introduction

The Transitional Care Unit (TCU) consortium has initiated the BRIDGE study to examine the effectiveness of the Jeroen Pit Huis (JPH). The JPH is a unique and innovative TCU situated in close proximity of the Amsterdam UMC. In this TCU, patients and their family reside in separate private home-like apartments as an intermediate step between hospital and home. The families can stay while practicing in, and adapting to, their new reality until they are ready to transition home. In this study, we are measuring differences in outcomes between patients discharges via the TCU JPH and patients discharged directly to home (control group). This includes patients from the Erasmus MC, Radboud umc, UMC Groningen and UMC Utrecht. Patients from the Amsterdam UMC who transition directly from hospital to home without an intermediate stay in the JPH will also be included in the control group.

In this blog, we explain how we will determine the effect of the intervention using bayesian inverse probability weighting methods. This blog only describes the analyses; the study protocol is registered at clinical trials.gov (hyperlink maken als nummer bekend is) and broader research design will be published in a scientific journal (if this is published, we will post them here)

Pictures of the Jeroen Pit Huis:

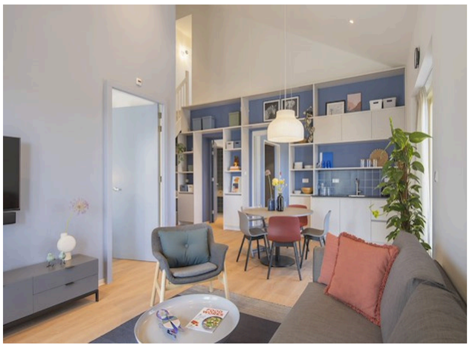

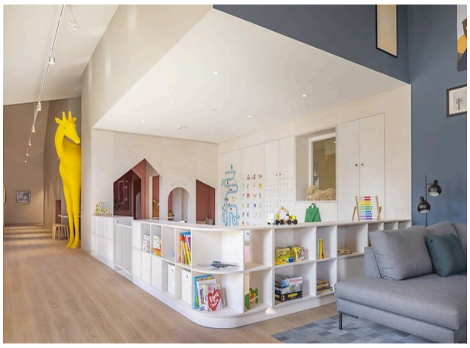

> **Note:** The BRIDGE study is currently ongoing and is expected to continue until at least mid-2025. Certain decisions we have made in this logbook are based on a previous study (H2H study - manuscript in preparation). The H2H study was conducted in preparation for the BRIDGE study due to the lack of existing data on the population of children in the Netherlands who potentially could use a TCU. In this study, the same population as in the BRIDGE study was included, with the objectives of: 1) describing the demographics, clinical characteristics, and course of patients transitioning from hospital to home; and 2) identifying perceived barriers to the postponement of hospital-to-home transitions. If final results are published, we will refer here to a new post including the results. This blog aimed at providing insights into the analyses.

2 Methodology

In this study, we have opted for Bayesian inverse probability weighting due to its suitability for our research objectives. In the following section, we'll delve into the rationale behind our methodological selection and elucidate its significant contribution to our analytically framework. Additionally, we will elucidate our data simulation process, detailing each step alongside the pertinent R code. We have gathered information and inspiration from diverse sources, including Huntington-Klein (2021), Heiss (2021a), and Heiss (2021b)

2.1 Why Bayesian analysis?

> **Note**: The text below is adapted from a previous blog. Read more about the principles of Bayesian analysis in that post.

We utilize Bayesian analysis principles to evaluate the efficacy of the JPH. This approach is adopted due to the constrained data points available in the BRIDGE study. Unlike traditional significance tests, which focus on determining whether an effect is statistically significant by testing how likely the observed data are conditional on the null hypothesis being true, our Bayesian analysis aims to estimate the probability that the intervention actually has a specific effect given the data. Bayesian methods are particularly beneficial for relatively small sample sizes because they allow the incorporation of prior information and provide more intuitive and interpretable results. Additionally, Bayesian analysis can produce credible intervals that directly reflect the uncertainty about parameter estimates, making it a robust choice for studies with limited data. In Bayesian analysis, we start by explicitly stating our expectation about the effect of the intervention. This initial belief is also known as a "prior." Although we theoretically expect parental stress and the length of hospital stay to decrease at the intervention practices, we have not yet seen evidence of this. Therefore, we formulate our prior conservatively and assume that the intervention has no effect, with a considerable degree of uncertainty. This means we initially assume that the chance of a positive effect (lower parental stress / fewer hospital days) is as great as a negative effect (higher parental stress / more hospital days).

Next, we collect data. Based on this data, we adjust our prior beliefs. The adjusted belief is expressed in the so-called 'posterior belief'. The posterior represents the probability that the intervention has an effect, taking into account both our initial belief (the prior) and the data. In other words: the posterior is a mix of the prior and the data. As we collect more data, the influence of the prior on the value of the posterior decreases (for example, we can get more data by adding intervention and control practices, or when we switch to an analysis at the patient level).

In summary, the use of Bayesian analysis allows us to work with limited data and still obtain useful insights about the likelihood of an intervention effect. This is particularly relevant in situations where it is difficult to collect enough data points to perform traditional significance tests. By shifting the focus from mere significance to probability, we can better deal with uncertainty and variability in our results.

2.2 Quasi-experimental study design

This study is a quasi-experimental prospective cohort study comparing the JPH to other H2H practices. We opted for this design because conducting a Randomized Controlled Trial (RCT) was not feasible. The assignment of patients to the intervention cannot be randomized because children are assigned to a ward/JPH based on the hospital. Geographical location determines a child's H2H program. Random assignment to specific hospitals is not feasible due to the practical difficulties and negative impact of relocating families from their usual living environment.

The question then remains: how to control confounding effects in this study? The problem is that populations between the intervention and control groups differ in characteristics and outcomes (confounding). In an RCT (Randomized Controlled Trial), study subjects are randomly assigned to exposure categories, which helps break any links between exposure and confounders. This random assignment reduces the potential for confounding. If these differences are not controlled for, the intervention effect may be underestimated or overestimated.

> **Example:** Imagine you want to measure the effect of an intervention that helps in getting a job. You want to compare a specific policy program in Rotterdam (intervention) with a existing policy program Amsterdam (control). However, in the intervention group, there are more women who already have a higher chance of getting a job than men. If we do not correct for gender, we may overestimate the intervention effect.

However, if an RCT is not feasible (as in our case), there are other options to estimate a causal effect. One of those methods is Difference in Differences (DiD) estimation. The idea behind a DiD is to compare the difference in outcome changes between the treatment group and the control group before and after the intervention. If the effect of the intervention is significant, this should be reflected in a larger difference in outcomes between the treatment and control groups after the implementation of the treatment, compared to before the implementation of the treatment. However, a DiD is also not feasible in our study because we do not have data available before the opening of the TCU. Therefore, we have opted for inverse probability weighting.

2.3 Inverse probability weigthing

To adjust for confounders we use bayesian inverse probability weighting. The following steps need to be undertaken:

1. Create a model that predicts treatment. Use confounders (identified with a Directed Acyclic Graph(DAG)) as the covariates. So: Predicts for each patient the probability that the patient - given their charateristics (confounders) - is in the treatment group. For this, we use Bayesian Logistic regressions (Bernoulli distribution). We refer to this probability as 'propensity scores'.

2. Generate samples of propensity scores based on the posterior distribution of propensity scores. This will give us a lot of propensity scores.

3. Convert those propensity scores into *inverse probability of treatment weights* (IPTW) using this formula:

$$\frac{\text{Treatment}}{\text{Propensity score}} + \frac{1 - \text{Treatment}}{1 - \text{Propensity score}}$$

4. Run an outcome model using those weights.

3 Datasimulation

Below we will elucidate our data simulation process, detailing each step alongside the pertinent R code. We use R version: 4.3.3.Ensure that the following four packages are downloaded.

▼ Code

```
# Load libraries (please install the packages if needed)

library(tidyverse) # for data manipulation and plotting
library(broom) # tidy regression output
library(ggdag) # nicer dags
library(brms) # for bayesian analysis
```

3.1 Directed Acyclic Graph (DAG)

Directed Acyclic Graphs (DAGs) are tools used to represent and analyze causal relationships in statistical models. They consist of nodes representing variables and directed edges indicating the direction of causality.DAGs help identify and control for confounding variables, making them useful for the construction of and communicating about causal models.

Pearl and Mackenzie (2018) provides a popular and accessible introduction to the concepts and applications of DAGs. It illustrates how these graphs can clarify causal questions and guide effective data analysis. See Pearl, Glymour, and Jewell (2016) for a more comprehensive and detailed treatment.

Our strategy involves mapping out the confounding factors using a directed acyclic graph (DAG) and then controlling for these differences. It is crucial to our approach that our DAG is both *accurate* and *complete*.

For this simulation we use a simplified generic DAG for two outcomes. Below in the DAG we show the relationship between getting the treatment (going to the JPH) (measured as a 0/1 binary variable where 1 = person went to the JPH) and Length of hospital stay (measured in days) / parental stress (measured on a scale of 0-10, with higher values representing higher amount of stress). Both are confounded by the number of children in the family, if the index patient is de oldest child in the family, is parents are living together, and if they are dutch speaking.

▼ Code

```
# Create DAG
JP_dag <- dagify(
  LOS ~ JP + Children + Eldest_child + Living_situation + Dutch,
  JP ~ Children + Eldest_child + Living_situation + Dutch,
  Eldest_child ~ Children,
  exposure = "JP",
  outcome = "LOS",
  coords = list(x = c(LOS = 7, JP = 3, Dutch = 4, Living_situation = 6, Children = 4, Eldest_child = 6),
                y = c(LOS = 2, JP = 2, Dutch = 1, Living_situation = 1, Children = 3, Eldest_child = 3)),
  labels = c(LOS = "Outcome\n(Length of Stay\nor Stress)", JP = "Jeroen Pit Huis", Children = "Number of ch
             Eldest_child = "Patient is eldest child", Living_situation = "Living situation",
             Dutch = "Dutch speaking"))

# Turn DAG into a tidy data frame for plotting
JP_dag1 <- JP_dag %>%
  tidy_dagitty() %>%
  node_status()   # Add column for exposure/outcome/latent

status_colors <- c(exposure = "#01796F", outcome = "#F79802", latent = "grey50")

# Fancier graph
ggplot(JP_dag1, aes(x = x, y = y, xend = xend, yend = yend)) +
  geom_dag_edges() +
  geom_dag_point(aes(color = status)) +
  geom_label(aes(label = label, fill = status),
             color = "white", fontface = "bold", nudge_y = -0.38, nudge_x = 0.3) +
  scale_color_manual(values = status_colors, na.value = "grey20") +
  scale_fill_manual(values = status_colors, na.value = "grey20") +
  guides(color = "none", fill = "none") +
  theme_dag()
```

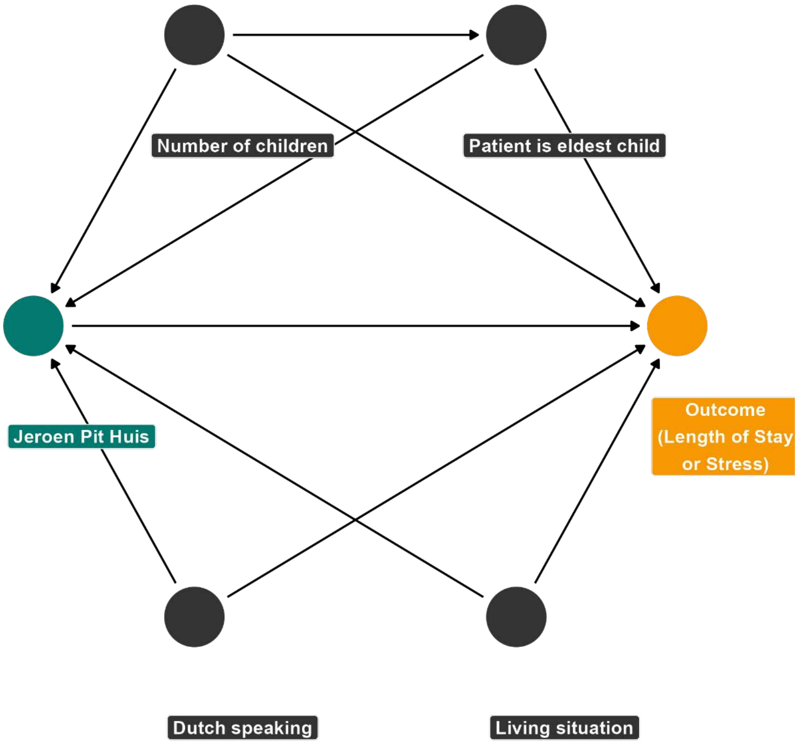

We simulate a data based on our DAG and the parameters of the H2H study mentioned in the introduction. This simulation involves generating data for patients from four different hospitals (AMC, EMC, RUMC, and UMCG) with varying characteristics such as living situations, language proficiency, number of children, and eldest patient status.

Specifically, we simulate:

1. **Living Situation**: Proportions of patients living together, alone, or in other arrangements.
2. **Language Proficiency**: Likelihood of patients speaking Dutch or other languages based on their hospital.
3. **Number of Children**: Distribution of patients having different numbers of children.
4. **Length of Stay (LOS)**: Estimated hospital stay length influenced by various factors such as hospital type, number of children, language proficiency, and living situation.
5. **Stress Levels**: Patient stress levels measured on a Likert scale, accounting for similar influencing factors. To generate the Likert scale for stress levels, we used latent variables, which are underlying factors that influence observed data but are not directly measured. In our simulation, we created a latent variable for stress and added specific influences from factors like eldest patient status, language proficiency, and living situation. We then mapped this latent variable onto a Likert scale (1-10) using predefined cut-off points.

▼ Code

```
set.seed(123)

# Settings: number of patients in 4 different hospitals
AMC <- 50  # AMC
EMC <- 60  # EMC
RUMC <- 40 # RUMC
UMCG <- 15 # UMCG

# Living situation proportions based on a total number of patients (Total_LS)
Total_LS <- 43
Together <- 34 / Total_LS
```

```
# Language proportions based on a total number of patients (Total_lang)
Total_lang <- 43
Dutch_AMC <- 7 / 10
Dutch_EMC <- 18 / 20
Dutch_RUMC <- 0.95
Dutch_UMCG <- 0.99

# Calculate the total number of patients
Total_patients <- sum(AMC, EMC, RUMC, UMCG)

# Effect sizes for length of stay (LOS) and stress
Effect_LOS <- 7
Effect_stress <- 0.2

# Cut-off points for latent variable to Likert scores (1-10)
cut_off <- c(-0.8, -0.6, 0.1, 0.5, 0.7, 0.9, 1, 1.1, 1.2)

# Mean and standard deviation for normally distributed variables
MU <- 1
SD <- 0.1

# Create the dataframe (df)
df <- tibble(ID = 1:Total_patients) %>%
  mutate(Hospital = as.factor(ifelse(ID <= AMC, "AMC",
                                     ifelse(ID <= AMC + EMC, "EMC",
                                            ifelse(ID <= AMC + EMC + RUMC, "RUMC", "UMCG"))))) %>%
  mutate(Treatment = as.factor(ifelse(Hospital == "AMC", 1, 0))) %>%
  mutate(T_num = as.numeric(as.character(Treatment))) %>%
  mutate(Living_situation = as.factor(sample(x = c("together", "alone", "other"), replace = TRUE,
                                             size = Total_patients, prob = c(Together, Alone, Other)))) %>%
  mutate(Dutch_speaking = as.factor(ifelse(Hospital == "AMC",
                                           sample(x = c("Dutch", "Non-Dutch"), replace = TRUE,
                                                  size = Total_patients, prob = c(Dutch_AMC, 1 - Dutch_AMC)
                                           ifelse(Hospital == "EMC",
                                                  sample(x = c("Dutch", "Non-Dutch"),
                                                         replace = TRUE, size = Total_patients,
                                                         prob = c(Dutch_EMC, 1 - Dutch_EMC)),
                                                  ifelse(Hospital == "RUMC",
                                                         sample(x = c("Dutch", "Non-Dutch"),
                                                                replace = TRUE, size = Total_patients,
                                                                prob = c(Dutch_RUMC, 1 - Dutch_RUMC)),
                                                         sample(x = c("Dutch", "Non-Dutch"),
                                                                replace = TRUE,
                                                                size = Total_patients, prob = c(Dutch_UMCG,
  mutate(NL = ifelse(Dutch_speaking == "Dutch", 0, 1)) %>%
  mutate(Number_of_children = ifelse(Hospital == "AMC", sample(x = c(1, 2, 3, 4, 5), size = Total_patients,
                                                               replace = TRUE, prob = c(0.5, 0.3, 0.1, 0.1,
                                     ifelse(Hospital == "EMC", sample(x = c(1, 2, 3, 4, 5), size = Total_pa
                                                                      replace = TRUE, prob = c(0.4, 0.35, 0
                                            ifelse(Hospital == "RUMC", sample(x = c(1, 2, 3, 4, 5), size =
                                                                              replace = TRUE, prob = c(0.37
                                                   sample(x = c(1, 2, 3, 4, 5), size = Total_patients,
                                                          replace = TRUE, prob = c(0.2, 0.4, 0.2, 0.1, 0.2)
  mutate(Eldest_patient = as.factor(ifelse(Number_of_children == 1, 1,
                                           ifelse(Hospital == "AMC", sample(x = c(0, 1), size = Total_patie
                                                                            prob = c(0.3, 0.7)),
                                                  sample(x = c(0, 1), size = Total_patients, replace = TRUE
                                                         prob = c(0.6, 0.4)))))) %>%
```

```
                      mean = 30 +
                        3 * Number_of_children +
                        1 * NL +
                        1 * Influence_living_situation +
                        1 * as.numeric(Eldest_patient), sd = 2)) %>%
  mutate(LOS = ifelse(Hospital == "AMC", LOS0 - Effect_LOS, LOS0)) %>%
  mutate(Stress_latent0 = rnorm(n = Total_patients, mean = MU - 0.3 * as.numeric(as.character(Eldest_patier
                                0.3 * NL -
                                0.3 * Influence_living_situation, sd = SD)) %>%
  mutate(Stress_latent = ifelse(test = Hospital == "AMC",
                                yes = Stress_latent0 + Effect_stress,
                                no = Stress_latent0)) %>%
  mutate(Stress0 = findInterval(Stress_latent0, cut_off) + 1) %>%
  mutate(Stress = findInterval(Stress_latent, cut_off) + 1) %>%
  mutate(Stress_fac = as.factor(Stress)) %>%
  mutate(Treatment_effect = Stress - Stress0)

df_CF <- df %>%
  mutate(Treatment = 0)

df_hospital <- df %>%
  select(id = ID, Hospital)
```

3.2 Visualization of the data (outcomes)

To better understand the distribution of our simulated data, we present histograms of the two main outcomes: length of stay (LOS) and stress levels. These visualizations help illustrate the variations and patterns within our synthetic data.

3.2.1 Length of Stay per hospital

▼ Code

```
ggplot(df, aes(x = LOS, fill = Hospital)) +
  geom_histogram(alpha = 0.5, binwidth = 3) +
  theme_bw() +
  labs(title = "Length of Stay in Days per Hospital", x = "", y = "") +
  facet_wrap(~ Hospital) +
  theme(legend.position = "none")
```

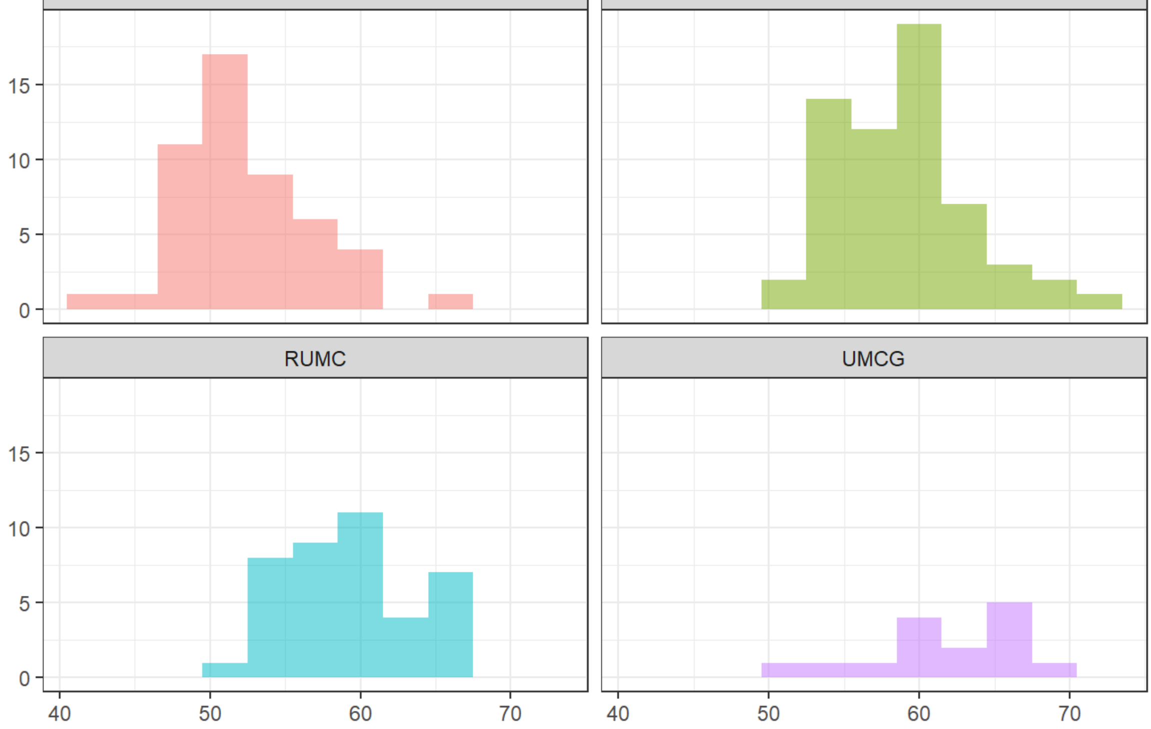

3.2.2 Stress levels per hospital

▼ Code

```
ggplot(df, aes(x = Stress, fill = Hospital)) +
  geom_histogram(binwidth = 1, alpha = 0.5) +
  theme_bw() +
  labs(title = "Stress per Hospital", x = "", y = "") +
  facet_wrap(~ Hospital) +
  scale_x_continuous(breaks = 1:10) +
  theme(legend.position = "none")
```

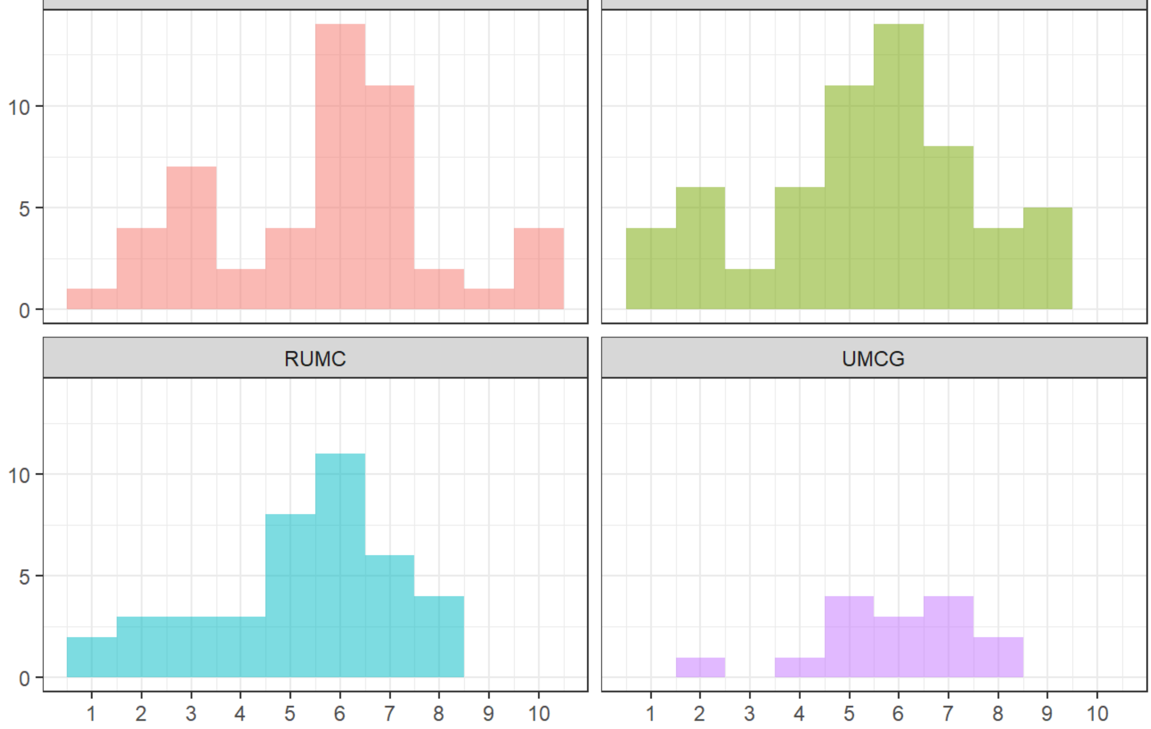

3.3 Analyse

Below the code how we calculate our propensity scores and IPTWs based on the confounders in our DAG:

▼ Code

```
fullrun <- 0 # saves time

if (fullrun) {

  model_prop_scores <- brm(
    formula = Treatment ~ Number_of_children + Eldest_patient + Living_situation + Dutch_speaking,
    family = bernoulli(),  # Logistic regression
    data = df,
    chains = 6,
    cores = 6,
    warmup = 500,
    iter = 1500,
    seed = 123,
    backend = "cmdstanr"
  )

  saveRDS(model_prop_scores, "model_prop_scores.RDS")

} else {

  model_prop_scores <- readRDS("model_prop_scores.RDS")

}
```

▼ Code

```
fullrun <- 0  # saves time

if(fullrun){

ipw_matrix <- t(pred_probs_chains) %>%
  as_tibble(.name_repair = "universal") %>%
  # Add treatment column so we can calculate weights
  mutate(T_num = df$T_num) %>%
  # Calculate weights
  mutate(across(starts_with("..."),
              ~ (T_num / .x) + ((1 - T_num) / (1 - .x))
  )) %>%
  # Get rid of treatment column
  select(-T_num)

saveRDS(ipw_matrix, "ipw_matrix.RDS")

} else {

ipw_matrix <- readRDS("ipw_matrix.RDS")

}

IPTW_help <- ipw_matrix %>%
  # Convert this matrix to a data frame
  as_tibble() %>%
  # Add a column for the draw number
  mutate(draw = 1:n()) %>%
  # Make this long so that each draw gets its own row
  pivot_longer(-draw, names_to = "row", values_to = "weights") %>%
  # Clean up the draw number
  mutate(draw = as.numeric(str_remove(row, "..."))) %>%
  select(-row)
```

3.4 Psuedo-populations

Before running the bayesian regressions, it’s helpful to understand what these weight are actually doing behind the scenes.

We can learn a few different things from the plot below. Fewer people received the treatment than didn’t—there are more people in the untreated part of the graph.

The two groups - intervention and control - aren’t comparable at this point. Therefore we created two pseudo-populations of treated and untreated people.Using the IPTW scores, individual patients are weighted, with patients having a higher likelihood of treatment exposure receiving less weight and patients with a lower likelihood receiving more weight. This results in a weighted dataset in which the confounding effects are reduced.

> **Note:** It’s challenging to visualize how we perform this using the Bayesian method because we have calculated 1500 propensity scores for each patient. To make it visualizable, we have chosen to take the mean of the propensity scores and then calculate the IPTWs based on the mean.

▼ Code

```
mean_propensity <- colMeans(pred_probs_chains)
        weights <- df %>%
  mutate(propensity = mean_propensity,
         iptw = (T_num / propensity) + ((1 - T_num) / (1 - propensity)))

# Plot the graph
ggplot() +
  geom_histogram(data = filter(df_with_weights, T_num == 1),
                 aes(x = propensity, weight = iptw), bins = 10,
                 fill = colorspace::lighten("#01796F", 0.35)) +
  geom_histogram(data = filter(df_with_weights, T_num == 0),
                 aes(x = propensity, weight = iptw, y = -after_stat(count)), bins = 10,
                 fill =  colorspace::lighten("#F79802", 0.35)) +
  geom_histogram(data = filter(df_with_weights, T_num == 1),
                 aes(x = propensity), bins = 10,
                 fill = "#01796F") +
  geom_histogram(data = filter(df_with_weights, T_num == 0),
                 aes(x = propensity, y = -after_stat(count)), bins = 10,
                 fill =  "#F79802") +
  geom_hline(yintercept = 0) +
  annotate(geom = "label", x = 1, y = 105, label = "Treated",
         fill = "#01796F", color = "white", hjust = 1) +
  annotate(geom = "label", x = 1, y = -80, label = "Untreated",
         fill = "#F79802", color = "white", hjust = 1) +
  annotate(geom = "label", x = 1, y = -100, label = "Untreated pseudo-population",
         fill = colorspace::lighten("#F79802", 0.35), color = "white", hjust = 1) +
  annotate(geom = "label", x = 1, y = 125, label = "Treated pseudo-population",
         fill = colorspace::lighten("#01796F", 0.35), color = "white", hjust = 1) +
  scale_y_continuous(label = abs) +
  labs(x = "Propensity", y = "Count") +
  coord_cartesian(xlim = c(0.0, 1.05)) +
  theme_bw()
```

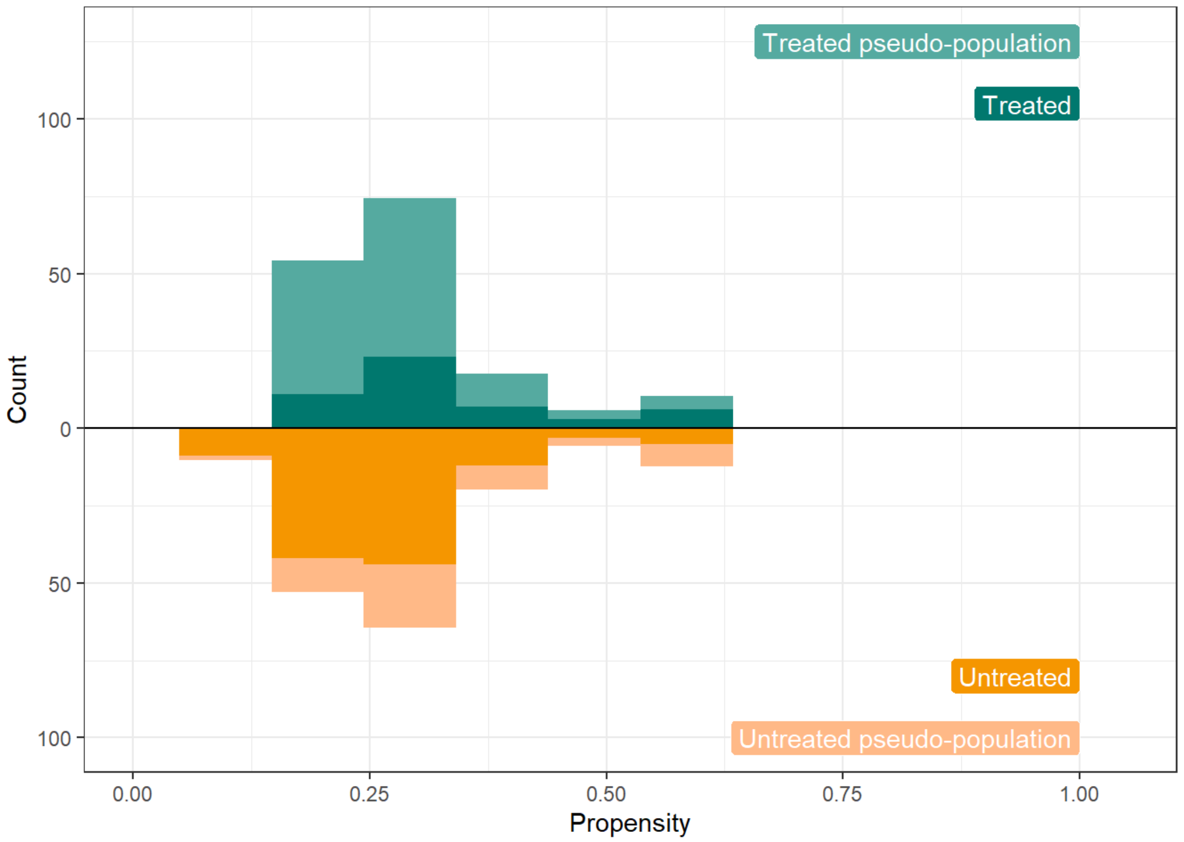

4 Analysis

In this section, we analyze the simulated data to estimate the causal effects of the Jeroen Pit Huis (JPH) intervention on patient outcomes, specifically focusing on length of hospital stay (LOS) and parental stress levels. Using the calculated inverse probability weights (IPWs), we conduct Bayesian regression analyses to adjust for confounders and provide robust estimates of the treatment effect. Given that we have a sample of the posterior distribution of the IPWs and considering that the brms package cannot directly take a matrix as input for weights, we performed 1500 regressions in a loop, each time using a different vector of weights. We begin with a Gaussian regression analysis for the numeric outcome variable LOS, followed by a cumulative model for the ordinal outcome variable of stress levels. The results of these regressions are presented as plots to visualize the estimated treatment effects and the uncertainty.

4.1 Length of hospital stay regression

To evaluate the impact of the JPH intervention on the length of hospital stay (a numeric variable and measured in days), we perform a Gaussian regression analysis.

Code

```
fullrun <- 0  # saves time

if (fullrun) {

  Number_regressions <- 1500  # moet 1500 zijn

  LDEstimate <- c()
  LDlower_95CI <- c()
  LDupper_95CI <- c()

  LDPreds <- tibble(id = 1:165)
  LDPreds_CF <- tibble(id = 1:165)

  for (i in 1:Number_regressions) {

     # Print the current iteration
    message("Running iteration: ", i)

    df_temp <- IPTW_help %>%
      filter(draw == i)

    df_temp2 <- df %>%
      bind_cols(df_temp)

    LDFormula <- LOS | weights(weights) ~ Treatment

    LDreg <- brm(
      formula = LDFormula,  # our formula
      data = df_temp2,  # data
      family = gaussian(),  # model for continuous data
      warmup = 500,
      iter = 1500,
      cores = 6,  # If you have 2 cores on your own computer set this to 2
      chains = 6,
      seed = 1729,
      silent = TRUE,
      backend = "cmdstanr"
    )
```

```
    LDlower_95CI[i] <- last_row[1, 3]
    LDupper_95CI[i] <- last_row[1, 4]

    LDResults <- tibble(LDEstimate, LDlower_95CI, LDupper_95CI)
  }

  saveRDS(LDResults, "LDResults.RDS")

} else {

  LDResults <- readRDS("LDResults.RDS")

}
```

▼ Code

```
ggplot(data = LDResults, aes(x = LDEstimate)) +
  geom_density(fill = "steelblue", alpha = 0.3) +
  theme_bw() +
  geom_vline(xintercept = -1 * Effect_LOS,
             color = "firebrick",
             linetype = "dashed",
             linewidth = 1) +
  labs(x = "Estimate of the treatment coefficient", y = "") +
  annotate(geom = "text", x = Effect_LOS * -1 + 0.7, y = 0.5,
           label = "Simulated effect",
           color = "firebrick") +
  xlim(-10, 10)
```

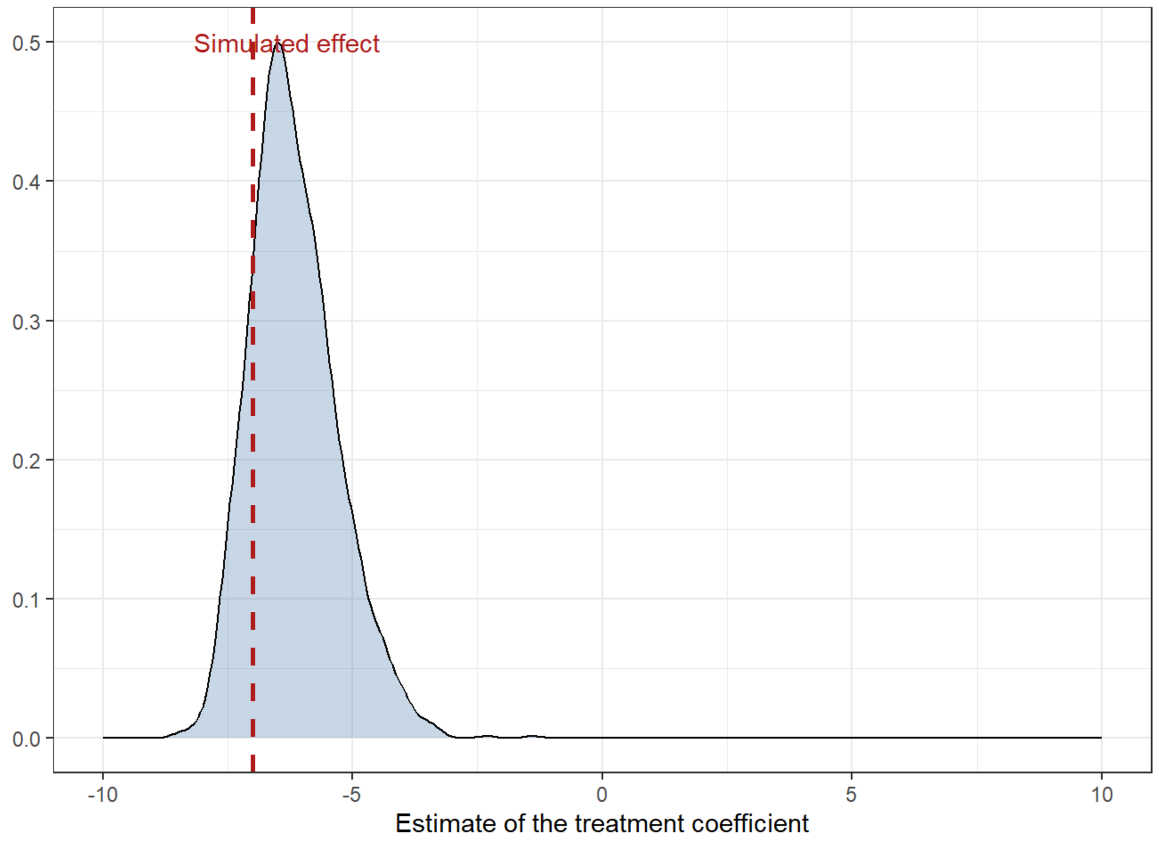

The plot above shows the distribution of the estimated treatment coefficients for the length of hospital stay (LOS) outcome. The vertical dashed line represents the simulated effect of the JPH intervention on LOS. The distribution of the estimated coefficients indicates the uncertainty around the treatment effect, with the majority of the estimates slightly overstating the simulated effect.

4.2 Parental stress regression

Next, we analyze the impact of the JPH intervention on parental stress levels, which are measured on an ordinal scale. We use a cumulative model to estimate the treatment effect on stress levels. The cumulative model estimates the probability that the stress level exceeds a certain threshold, given the treatment and control conditions.

▼ Code

```
Number_regressions <- 1500  # moet 1500 zijn
fullrun <- 0 # saves time

if(fullrun){





Estimate <- c()
lower_95CI <- c()
upper_95CI <- c()




for (i in 1:Number_regressions){

df_temp <- IPTW_help %>%
  filter(draw == i)

df_temp2 <- df %>%
  bind_cols(df_temp)

Formula <- Stress | weights(weights) ~ Treatment

reg <- brm(
  formula = Formula,    # onze formule
  data = df_temp2,              # data
  family = cumulative("probit"), # model voor ordinal data
  warmup = 500,
  iter = 1500,
  cores = 6, #If you have 2 cores on your own computer set this to 2
  chains = 6,
  seed = 1729,
  silent = TRUE,
  backend = "cmdstanr"

)

last_row <- tail(fixef(reg),1)

Estimate[i] <- last_row[1,1]
lower_95CI[i] = last_row[1,3]
upper_95CI[i] = last_row[1,4]

Results <- tibble(Estimate, lower_95CI, upper_95CI)

}
```

```
} else {

Results <- readRDS("Results.RDS")

}

Results <- Results %>%
  mutate(Iteration = 1:Number_regressions)
```

▼ Code

```
ggplot(Results, aes(x = Estimate)) +
  geom_histogram(aes(y = after_stat(density)), binwidth = 0.05, fill = "steelblue", alpha = 0.7) +
  geom_density(color = "steelblue4", linewidth = 1) +
  labs(x = "Estimate", y = "Density") +
  geom_vline(aes(
    xintercept = round( mean(df$Stress[df$Hospital == "AMC"]) - mean(df$Stress[df$Hospital != "AMC"]),2)),
    color = "firebrick",
    linetype = "dashed",
    linewidth = 1) +
  annotate(geom = "text", x = round( mean(df$Stress[df$Hospital == "AMC"]) - mean(df$Stress[df$Hospital !=
           label = "Simulated effect\nin the data",
           color = "firebrick") +
  theme_bw()
```

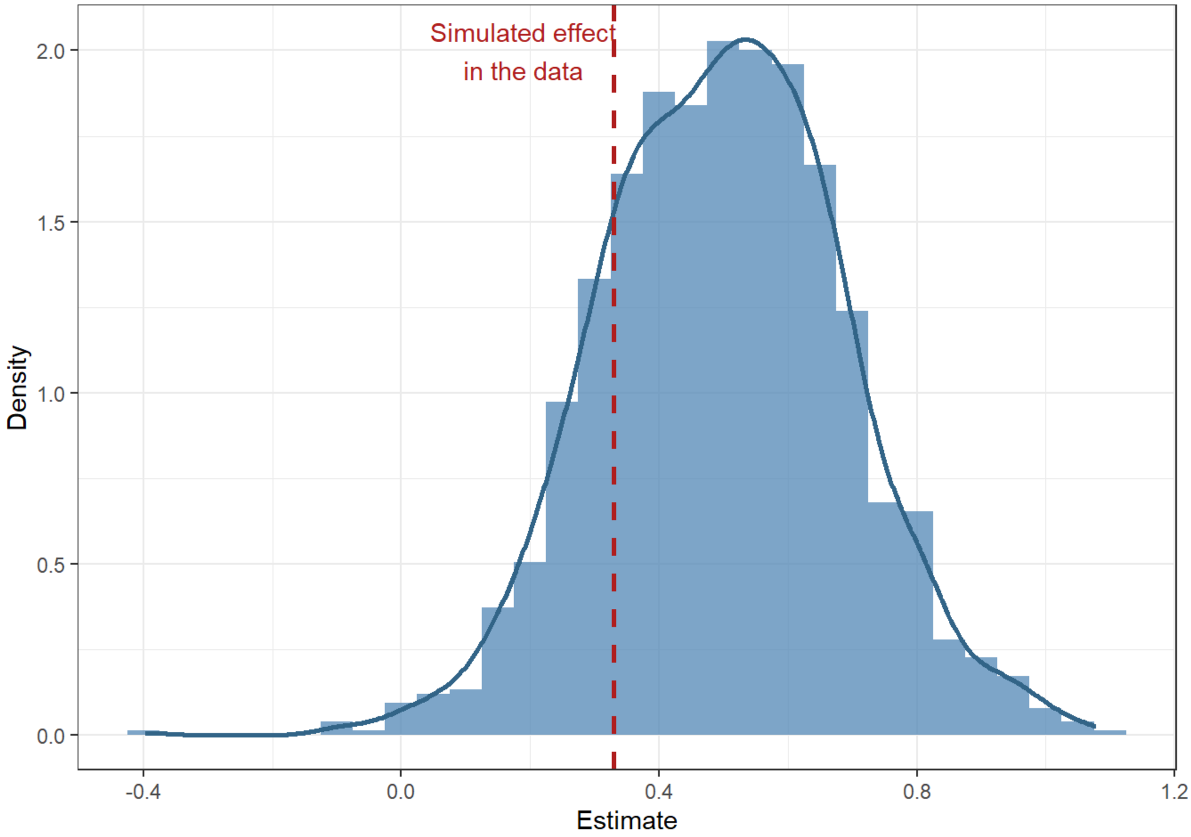

The plot above shows the distribution of the estimated treatment coefficients for the parental stress outcome. The vertical dashed line represents the simulated effect of the JPH intervention on parental stress levels. The distribution of the estimated coefficients indicates the uncertainty around the treatment effect, with the majority of the estimates overstating the effect to some extend.

5 Conclusion

As this blog serves as an online appendix for our study protocol, it provides insight into the methods we will use in our evaluation of the Jeroen Pit Huis (JPH) intervention. It is important to note that this blog and analysis have been conducted without access to the actual study data.We had access to the descriptive data in the -to be submitted- H2H study. The data was simulated to mimic the actual data.

Our analysis of the simulated data demonstrates the application of our chosen methods that we will use to estimate the effects of the JPH intervention on patient outcomes. For the length of hospital stay (LOS), the Bayesian Gaussian regression analysis demonstrated that the majority of the estimated treatment effects slightly overstate the simulated effect, reflecting a degree of uncertainty inherent in our estimates. The simulated effect size for LOS was 7, while the average effect size estimated from the data was approximately -6.17.

Similarly, the cumulative model for parental stress levels indicates an overestimation of the effect size as well. The average effect size for parental stress was approximately 0.5, compared to the effect size observed in the data, which was 0.33. The initial effect included in the simulation was 0.2. The slight overestimation observed in our model can be attributed to several factors, including sampling variability due to the small sample size and the non-linearity introduced by translating latent stress to ordinal scores.

Overall, our Bayesian approach, combined with inverse probability weighting, helps us manage the challenges of a limited sample size and noisy data, where traditional significance testing might be less effective.

6 References

Heiss, Andrew. 2021a. “How to Use Bayesian Propensity Scores and Inverse Probability Weights.” December 18, 2021. https://doi.org/10.59350/nrwsd-3jz20.

———. 2021b. “How to Create a(n Almost) Fully Bayesian Outcome Model with Inverse Probability Weights.” December 20, 2021. https://doi.org/10.59350/gyvjk-hrx68.

Huntington-Klein, Nick. 2021. *The Effect: An Introduction to Research Design and Causality*. Chapman; Hall/CRC.

Pearl, Judea, Madelyn Glymour, and Nicholas P Jewell. 2016. *Causal Inference in Statistics: A Primer*. John Wiley & Sons.

Pearl, Judea, and Dana Mackenzie. 2018. *The Book of Why: The New Science of Cause and Effect*. Basic books.
