# Supplementary material for: Bridging hospital to home for children with medical complexity and their families: an observational prospective cohort study protocol to assess the effectiveness of an innovative transitional care unit in the Netherlands (BRIDGE study)
Source: BMJ Open. 2025 Apr 19;15(4):e093693. doi: 10.1136/bmjopen-2024-093693 (PMC12010278; doi:10.1136/bmjopen-2024-093693)
Supplement: Supplementary file 3 [file bmjopen-2024-093693.reviewer_comments.pdf]

PEER REVIEW HISTORY

BMJ Open publishes all reviews undertaken for accepted manuscripts. Reviewers are asked to complete a checklist review form and are provided with free text boxes to elaborate on their assessment. These free text comments are reproduced below.

ARTICLE DETAILS

Title (Provisional)

Bridging hospital to home for children with medical complexity and their families: an observational prospective cohort study protocol to assess the effectiveness of an innovative Transitional Care Unit in the Netherlands (BRIDGE study)

Authors

Haspels, Heleen; Mikkers, M.C.; Knoester, H.; Jansen, N.J.G.; Ahout, Inge; Maaskant, J.M.; van de Riet , L.; Haverman, Lotte; Dulfer, K.; Haasnoot, M.; Alsem, Mattijs; De Hoog, Matthijs; van Woensel, Job; Joosten, K; van Karnebeek, Clara; Consortium, Transitional Care Unit

---

VERSION 1 - REVIEW

**Reviewer**	**1**
**Name**	**Ang, Ian**
**Affiliation**	**National University Singapore Saw Swee Hock School of Public Health**
**Date**	**19-Nov-2024**
**COI**	**None**

This study assesses a real-world intervention for paediatric patients with complex medical conditions, JPH, which is a transition care unit that mimics a home setting. I applaud the study team and clinicians in rolling out this meaningful intervention and assessing it in a systematic fashion, presented in a well-written manuscript. I look forward to the results when the study concludes!

Here are some very minor comments to improve the manuscript:

Page 6 Line 32: “5-pointLikert scale” should have a space

Page 6 Line 33 and Line 41: “mean 50” – should be rephrased as “mean of 50” or “mean = 50”

Page 6 Line 33 and Line 41: “SD10” – SD should be defined and perhaps equal sign be included e.g. “Standard Deviation (SD) = 10”

Page 6 Line 40-41: “a standardized T scores” – score should be singular to be consistent.

**Reviewer**	**2**
**Name**	**Arp Sørensen, Charlotte**
**Affiliation**	**Hospital Pharmacy Central Denmark Region, Research & Development**
**Date**	**07-Jan-2025**
**COI**	**None**

Thank you for letting me review this paper (Bridging hospital to home for children with medical complexity and their families: an observational prospective cohort study protocol to assess the effectiveness of an innovative Transitional Care Unit in the Netherlands (BRIDGE study)).

It is an interesting study. I appreciate your interest in bridging the transition from hospital to home as this may be problematic for these families.

There are many questionnaires for the parents. I would have been interesting to make a health economic analysis to see if the TCU is cost-effective. E.g. a cost-consequence analysis as there may be more outcomes/consequence of interest.

Using mixed-methods could perhaps have given you additional understandings on the phenomenon.

Good luck.

Comments

In general: The manuscript is well-written and I have only a few comments.

Abstract

Introduction, you write: ”We hypothesize that an intermediate stay at the JPH will have a favorable effect on healthcare consumption, patient, parent and family-relevant outcomes in comparison to discharge directly from a hospital ward to home. We expect that an intermediate stay at the JPH, serving as a step between hospital and home, will positively impact healthcare consumption and outcomes relevant to patients, parents, and families, in comparison to direct discharge from a hospital ward to home”.

To me, it seems like the two sentences says the same. Is it an unnecessary repetition?

Also you write: “We hypothesize”…. “We expect”…..

I prefer to have the “purpose” more clear…..”Therefore, the purpose of this study is to…..”

Please consider refrasing. (Just take the words from your description of the purpose in the introduction)

Under methods/analysis I miss some information on the intervention. What does it mean to be transitioned through the TCU? Later in the paper, you explain it so well. Is there room for some elaboration within the abstract as well?

E.g. “During the stay, more and more responsibilities and tasks are gradually transferred to

the parents until they become the primary caregivers. This process is guided by a newly developed

structured care pathway, which is informed by the parental needs”

Please consider elaborating on the intervention.

Introduction

p. 3, l. 11. “…revealed that parents experience and express (unmet) needs across several domains, including learning complex nursing care…”. Why do you put unmet in brackets ()? I find the brackets confusing. Please consider to remove the brackets.

p.2, l.31: “…Amsterdam UMC…” What does the abbreviation UMC stand for? Please elaborate. It is explained on p.4.

p. 2, l. 32: “…Families can stay together while practising in, and adapting to, their new reality until they are ready to transition home.” How long is their stay? Hours, days or weeks?

p. 2, l.31-39: I miss information about the intervention. What is the task for each of the professionals? Does Pharmacy personel have any role?

Method

Both care facilities and structured care pathway.

p.4, l.34: A care pathway is mentioned with a reference (in which eight themes are mentioned). You also write “It is organized into seven phases, each with a step-by-step transition plan”. Is it possible to elaborate these phases e.g. in a figure? It is hard to understand the intervention besides living in a care facility without this information. Please elaborate.

p. 8, l. 12: You mention a self-conducted survey on financial impact. How was the survey developed/conducted?

p.8, l. 15. You refer to supplementary 6: Please check Table 1 as you here refer to suppl 7.

p. 11, l. 20: You describe the sample size and mention that the goal is to recruit 50 in the intervention group and 100 in the control group.

Have you made a calculation of the sample size based on the primary outcome or is it just what you consider to be the number possible to recruit during the study period?

Can you explain why you want to recruit 100 instead of 50 in the control group?

**Reviewer**	**3**
**Name**	**Halm, Josiah**
**Affiliation**	**The University of Texas MD Anderson Cancer Center, Hospital Medicine**
**Date**	**24-Jan-2025**
**COI**	**None**

1. Was the JPU unit built to address H2H transition?

2. Are the UMC's similar in atheir ability to care for CMC patients?

3. Cost of care - Is it cheaper to step down patient to JPU?

4. address capacity issues for JPU to meet eligible patient due to lack of space

VERSION 1 - AUTHOR RESPONSE

Reviewer # 1:
This study assesses a real-world intervention for paediatric patients with complex medical conditions, JPH, which is a transition care unit that mimics a home setting. I applaud the study team and clinicians in rolling out this meaningful intervention and assessing it in a systematic fashion, presented in a well-written manuscript. I look forward to the results when the study concludes!

Here are some very minor comments to improve the manuscript.
- Page 6 Line 32: "5-pointLikert scale" should have a space
- Page 6 Line 33 and Line 41: "mean 50" – should be rephrased as "mean of 50" or "mean = 50"
- Page 6 Line 33 and Line 41: "SD10" – SD should be defined and perhaps equal sign be included e.g. "Standard Deviation (SD) = 10"
- Page 6 Line 40-41: "a standardized T scores" – score should be singular to be consistent.

Author's response:
We sincerely appreciate the reviewer's thoughtful feedback and kind words about our study. We are grateful for the recognition of our work in evaluating this real-world intervention and look forward to sharing the study results in due course.

In response to the minor comments:
- We have added a space in "5-point Likert scale" (Page 6, line 23)
- We have revised "mean 50" to "mean of 50" (Page 6, line 24)
- We have defined SD as "Standard Deviation (SD)" and revised the notation to "SD of 10" (Page 6, lines 24-25)
- We have rewritten the sentence as: *"The raw score will be converted into a T-score with a mean of 50 and SD of 10"* for consistency (Page 6, lines 30-31)

Reviewer # 2:

Thank you for letting me review this paper (Bridging hospital to home for children with medical complexity and their families: an observational prospective cohort study protocol to assess the effectiveness of an innovative Transitional Care Unit in the Netherlands (BRIDGE study)).

It is an interesting study. I appreciate your interest in bridging the transition from hospital to home as this may be problematic for these families.

There are many questionnaires for the parents. I would have been interesting to make a health economic analysis to see if the TCU is cost-effective. E.g. a cost-consequence analysis as there may be more outcomes/consequence of interest.

Using mixed-methods could perhaps have given you additional understandings on the phenomenon.

Good luck.

Author's response:

We sincerely appreciate the reviewer's thoughtful feedback and kind words about our study. Thank you for recognizing the importance of bridging the transition from hospital to home for children with medical complexity and their families.

Regarding the suggestion for a health economic analysis, we deliberately chose not to include cost-effectiveness questionnaires in this study, as parents were already asked to complete multiple surveys. However, we are assessing outcomes such as length of stay and readmission rates, which are key indicators of healthcare resource utilization and may provide indirect insights into cost implications. A comprehensive evaluation of the TCU's cost-effectiveness requires a separate study, as this falls outside the scope of our current research.

We fully agree with the reviewer that a mixed-methods approach could provide additional insights into the phenomenon. In addition to the current quantitative study, we have also conducted a qualitative evaluation of the care provided in the TCU, including interviews with parents. This study is currently under revision. Furthermore, we are conducting a mixed-methods process evaluation of the TCU's implementation.

Comment 1 (abstract):

Introduction, you write*: "We hypothesize that an intermediate stay at the JPH will have a favorable effect on healthcare consumption, patient, parent and family-relevant outcomes in comparison to discharge directly from a hospital ward to home. We expect that an intermediate stay at the JPH, serving as a step between hospital and home, will positively impact healthcare consumption and outcomes relevant to patients, parents, and families, in comparison to direct discharge from a hospital ward to home".* To me, it seems like the two sentences says the same. Is it an unnecessary repetition? Also you write: "We hypothesize"…. "We expect"….. I prefer to have the "purpose" more clear….."Therefore, the purpose of this study is to….." Please consider refrasing. (Just take the words from your description of the purpose in the introduction)

Author's response:

We agree with the reviewer that the two sentences were somewhat repetitive. We have revised this section to clearly state the purpose of the study, aligning it with the description in the introduction (Page 2, lines 10-12).

Comment 2 (abstract):

Under methods/analysis I miss some information on the intervention. What does it mean to be transitioned through the TCU? Later in the paper, you explain it so well. Is there room for some elaboration within the abstract as well?

E.g. "During the stay, more and more responsibilities and tasks are gradually transferred to

the parents until they become the primary caregivers. This process is guided by a newly developed structured care pathway, which is informed by the parental needs”
Please consider elaborating on the intervention.

Author’s response:
We agree that the description of the intervention needed to be more clearly highlighted in the abstract. We have now added that families can stay together in a home-like apartment under the supervision of healthcare professionals. For readers who want more details, the intervention is further elaborated on later in the manuscript (Page 2, lines 6-8).

Comment 3 (introduction):
p. 3, l. 11. “…revealed that parents experience and express (unmet) needs across several domains, including learning complex nursing care…”. Why do you put unmet in brackets ()? I find the brackets confusing. Please consider to remove the brackets.

Author’s response:
We included the brackets around “unmet” to indicate that while parents express needs across several domains, these needs are not always unmet—some are adequately addressed, while others are not. However, we understand that the brackets may be confusing, so we have reconsidered the phrasing for clarity (Page 3, line 7-8) .

Comment 4 (introduction):
p.2, l.31: “…Amsterdam UMC…” What does the abbreviation UMC stand for? Please elaborate. It is explained on p.4.

Author’s response:
UMC stands for University Medical Center. We have added this explanation in the manuscript at its first mention on page 2 for clarity (Page 3, line 23).

Comment 5 (introduction):
p. 2, l. 32: “…Families can stay together while practising in, and adapting to, their new reality until they are ready to transition home.” How long is their stay? Hours, days or weeks?

Author’s response:
The length of stay varies per family, but the general guideline is a minimum of one week and a maximum of three months. We have added this information to the manuscript (Page 3, lines 25-26).

Comment 6 (introduction):
p. 2, l.31-39: I miss information about the intervention. What is the task for each of the professionals? Does Pharmacy personel have any role?

Author’s response:
We agree that more details about the intervention, including the roles of different professionals, are important. However, we believe this information fits better in the Methods section rather than the Introduction. To clarify, we have now explicitly stated which professionals are employed by the TCU and with whom we collaborate, such as pharmacy personnel in both the hospital and home settings (page 4, lines 20-22).

Comment 7 (method):
Both care facilities and structured care pathway.

p.4, l.34: A care pathway is mentioned with a reference (in which eight themes are mentioned). You also write “It is organized into seven phases, each with a step-by-step transition plan”. Is it possible to elaborate these phases e.g. in a figure? It is hard to understand the intervention besides living in a care facility without this information. Please elaborate.

Author’s response:
We sincerely appreciate the reviewer’s suggestion to elaborate on the seven-phase structured care pathway. To enhance clarity, we have added a more detailed description of these seven phases in Appendix 1, which provides a more comprehensive understanding of the structured approach beyond the physical care facility itself. Of note, we are currently developing a national Dutch TCU clinical care pathway, which will include more details and consensus-based recommendations for providing hospital-to-home care for children with medical complexity.

Comment 8 (method):
p. 8, l. 12: You mention a self-conducted survey on financial impact. How was the survey developed/conducted?

Author’s response:
The questions for the survey on financial impact were developed by the authors and discussed with consortium members, including a parent representative, to ensure relevance and comprehensibility. We added this to the manuscript (page 7, 8-9).

Comment 9 (method):
p.8, l. 15. You refer to supplementary 6: Please check Table 1 as you here refer to suppl 7.

Author’s response:
You are correct—it should indeed be Supplement 6. We have corrected this reference in Table 1 accordingly.

Comment 10 (method):
p. 11, l. 20: You describe the sample size and mention that the goal is to recruit 50 in the intervention group and 100 in the control group. Have you made a calculation of the sample size based on the primary outcome or is it just what you consider to be the number possible to recruit during the study period? Can you explain why you want to recruit 100 instead of 50 in the control group?

Author’s response:
As stated under the Sample Size section, our goal is to include as many patients as possible within the 1.5-year study period, and we have not conducted a formal sample size calculation. Instead, we have assessed whether the expected inclusion numbers would allow us to detect the anticipated effects. This pragmatic approach was chosen to ensure feasibility within the study timeline while still enabling meaningful analysis of the intervention's impact.

Reviewer #3:
Comment 1:
Was the JPU unit built to address H2H transition?

Author’s response:
Yes, the JPH was specifically established to improve H2H transitions for CMC and their families (Page 3, lines 20-22).

Comment 2:
Are the UMC's similar in atheir ability to care for CMC patients?

Author's response:
The UMCs all provide specialized care for CMC patients and all of these hospitals are equipped with a pediatric intensive care unit, and various general pediatric departments. Since there is no standardized care path, the H2H transitions vary across the different UMCs and even within different departments. There are no other TCUs in the Netherlands like the JPH. (page 4, lines 38-40)

Comment 3:
Cost of care - Is it cheaper to step down patient to JPU?

Author's response:
A formal cost-effectiveness analysis is not included in this study, but JPH may reduce healthcare costs by shortening hospital stays and preventing avoidable readmissions. However, a full economic evaluation is needed to quantify these potential cost savings.

Comment 4:
Address capacity issues for JPU to meet eligible patient due to lack of space

Author's response:
To date, we have not experienced capacity issues at JPH. However, if capacity limitations arise, eligible patients who cannot be admitted will be included in the control group at Amsterdam UMC. We will address this in the final manuscript.

VERSION 2 - REVIEW

**Reviewer**	**2**
**Name**	**Arp Sørensen, Charlotte**
**Affiliation**	**Hospital Pharmacy Central Denmark Region, Research & Development**
**Date**	**26-Mar-2025**
**COI**	

Thank you for revising the manuscript. I can see that you have carefully gone through reviewer comments and revised accordingly.

VERSION 2 - AUTHOR RESPONSE

Reviewer: 2
Dr. Charlotte Arp Sørensen, Hospital Pharmacy Central Denmark Region
Thank you for revising the manuscript. I can see that you have carefully gone through reviewer comments and revised accordingly. Good luck.

Author’s response:
We sincerely thank Reviewer 2 for taking the time to review our revised manuscript and for the kind and encouraging feedback. We appreciate your positive assessment of our revisions.
